# Two enzymes with redundant fructose bisphosphatase activity sustain gluconeogenesis and virulence in *Mycobacterium tuberculosis*

**DOI:** 10.1038/ncomms8912

**Published:** 2015-08-10

**Authors:** Uday Ganapathy, Joeli Marrero, Susannah Calhoun, Hyungjin Eoh, Luiz Pedro Sorio de Carvalho, Kyu Rhee, Sabine Ehrt

**Affiliations:** 1 Department of Microbiology and Immunology, Weill Cornell Medical College, 413 East 69th Street, New York, New York 10021, USA; 2 Department of Medicine, Weill Cornell Medical College, New York, New York 10021, USA; 3 Francis Crick Institute, Mill Hill Laboratory, The Ridgeway, Mill Hill, London NW71AA, UK

## Abstract

The human pathogen *Mycobacterium tuberculosis* (*Mtb*) likely utilizes host fatty acids as a carbon source during infection. Gluconeogenesis is essential for the conversion of fatty acids into biomass. A rate-limiting step in gluconeogenesis is the conversion of fructose 1,6-bisphosphate to fructose 6-phosphate by a fructose bisphosphatase (FBPase). The *Mtb* genome contains only one annotated FBPase gene, *glpX*. Here we show that, unexpectedly, an *Mtb* mutant lacking GLPX grows on gluconeogenic carbon sources and has detectable FBPase activity. We demonstrate that the *Mtb* genome encodes an alternative FBPase (GPM2, Rv3214) that can maintain gluconeogenesis in the absence of GLPX. Consequently, deletion of both GLPX and GPM2 is required for disruption of gluconeogenesis and attenuation of *Mtb* in a mouse model of infection. Our work affirms a role for gluconeogenesis in *Mtb* virulence and reveals previously unidentified metabolic redundancy at the FBPase-catalysed reaction step of the pathway.

## Abstract (web summary)

*Mycobacterium tuberculosis* feeds on host fatty acids during infection, a process that requires a fructose bisphosphatase (FBPase) enzyme for gluconeogenesis. Here, Ganapathy *et al.* show that the bacterium has two different FBPases and that this enzymatic activity is required for full virulence.

## Graphical abstract

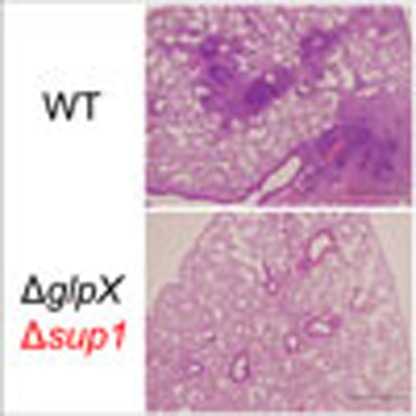

M*ycobacterium tuberculosis* (*Mtb*) is a resilient intracellular bacterium capable of infecting and surviving within host macrophages. *Mtb*’s ability to persist and ultimately establish a latent infection requires energy and biomass. Therefore, *Mtb* carbon metabolism is critical to the pathogen’s virulence and represents a new area for tuberculosis (TB) drug development. Our understanding of the enzymes and metabolic pathways that contribute to *Mtb*’s pathogenicity, however, remains incomplete.

The unique nature of *Mtb* carbon metabolism was reflected in an early observation that *Mtb* recovered from infected animals preferentially respires fatty acids instead of glycolytic substrates^1^,^2^. A mounting body of evidence now suggests that fatty acids are a significant carbon source for *Mtb* during an infection^3^. Following beta-oxidation, carbon from fatty acids can be readily directed towards the TCA cycle for energy production. Alternatively, carbon flux can be routed towards biomass production via gluconeogenesis, a pathway that generates glucose 6-phosphate (G6P) and fructose 6-phosphate (F6P), precursors for nucleotide and cell wall biosynthesis, respectively (Fig. 1). Thus, gluconeogenesis is critical to *Mtb*’s ability to convert fatty acids into biomass and is likely required for the pathogen to cause disease.

Early evidence for a role of gluconeogenesis in *Mtb* virulence comes from studies of isocitrate lyase ICL, an enzyme that operates in the glyoxylate shunt. Biomass production from fatty acids requires the glyoxylate shunt to bypass the oxidative branch of the TCA cycle, where carbon is lost as CO_2_, and enable gluconeogenic carbon flow. Loss of ICL abolished growth of *Mtb* on fatty acids *in vitro* and led to early clearance of the pathogen from the lungs of infected mice^4^. These phenotypes were attributed to a requirement for gluconeogenesis, as disruption of the glyoxylate shunt blocks the flow of carbon from fatty acids into this pathway. This interpretation, however, does not account for the fact that ICL also functions as a methylisocitrate lyase in the methylcitrate cycle^5^. Besides inhibiting gluconeogenesis, loss of methylisocitrate lyase activity in ICL-deficient *Mtb* also perturbed *Mtb’s* intrabacterial pH and membrane potential^6^. Given the pleiotropic effects of ICL deletion, it is unclear to what extent the ICL mutant’s *in vivo* growth and survival defects are caused by the interruption of gluconeogenesis.

Phosphoenolpyruvate carboxykinase (PEPCK), encoded in *Mtb* by *pckA,* catalyses the first committed step of gluconeogenesis, converting oxaloacetate into phosphoenolpyruvate. Loss of PEPCK in *Mtb* results in a block of gluconeogenesis, but the enzyme is dispensable for glycolysis^7^. Similar to ICL-deficient *Mtb*, *Mtb* lacking PEPCK cannot utilize fatty acids to support *in vitro* growth and fails to establish and maintain infection in mouse lungs. Thus, the inability of PEPCK-deficient *Mtb* to survive *in vivo* is associated with a disruption of gluconeogenesis that renders the bacterium unable to utilize host fatty acids. However, PEPCK has been reported to also operate in the reverse direction, converting phosphoenolpyruvate into oxaloacetate to facilitate pyruvate distribution in metabolism under conditions of slowed growth^8^. PEPCK’s anaplerotic activity brings into question whether the *in vivo* survival defect of PEPCK-deficient *Mtb* is solely due to disrupted gluconeogenesis.

Fructose bisphosphate aldolase (FBA) and triose phosphate isomerase (TPI) are also required for *Mtb* to grow on fatty acids *in vitro*, and mutants lacking these enzymes are attenuated *in vivo*^9^,^10^. However, the reactions catalysed by these enzymes are bidirectional; loss of either FBA or TPI disrupts both gluconeogenesis and glycolysis. Thus, the extent to which loss of gluconeogenesis, as opposed to disruption of glycolysis, contributes to the *in vivo* survival defect of FBA and TPI mutants cannot be determined. The question of whether gluconeogenesis is required for *Mtb* virulence remains unanswered.

In this work, we sought to address the role of gluconeogenesis in *Mtb* virulence by studying fructose bisphosphatase (FBPase). FBPases catalyse the rate-limiting step of gluconeogenesis in which fructose 1,6-bisphosphate (FBP) is hydrolysed to yield F6P and inorganic phosphate. Five classes of FBPases have been defined based on primary sequence. Eukaryotes only encode the Type I FBPase, while all five types can be found among prokaryotes. Types I, II and III FBPases are expressed in bacteria^11^,^12^,^13^,^14^, while the Type IV enzyme is found primarily in archaea^15^. Type V FBPases are FBP aldolase/phosphatases that are associated with thermophilic prokaryotes from both archaea and bacteria^16^,^17^,^18^,^19^,^20^. Unlike other steps in gluconeogenesis, the FBPase reaction is unidirectional and specific to this pathway. Thus, ablation of FBPase activity will only disrupt gluconeogenesis while leaving glycolysis unperturbed, allowing for a direct assessment of the specific role of gluconeogenesis in *Mtb* virulence.

*GlpX* (*rv1099c*) encodes the only annotated FBPase in the *Mtb* genome (Fig. 1). GLPX is classified as a Type II FBPase based on its homology to an *Escherichia coli* FBPase of the same name and belongs to the metal-dependent/Li^+^-inhibited phosphomonoesterase protein family^21^. The annotation of GLPX as an FBPase was validated by the demonstration that overexpression of this enzyme rescued growth and FBPase activity of an *E. coli* FBPase mutant on a gluconeogenic carbon source^22^. Furthermore, recombinant GLPX has FBPase activity with a reported *K*_m_ of 44 μM and a *k*_cat_ of 1.0 s^−1^ (ref. 23). To study the role of FBPase and gluconeogenesis in *Mtb* virulence, we generated a *glpX* deletion mutant (Δ*glpX*). In agreement with previous TraSH and TnSeq analyses^24^,^25^, we found that *glpX* is not essential for *in vitro* growth of *Mtb* on glycerol and fatty acids. We demonstrate that GPM2, a broad-specificity phosphatase^26^, has FBPase activity that maintains *Mtb* gluconeogenesis in the absence of GLPX. Only deletion of both FBPases disrupted gluconeogenesis and rendered *Mtb* unable to establish infection, affirming the importance of this pathway to virulence.

## Results

### GLPX is dispensable for growth on gluconeogenic carbon sources

**Figure 2:**
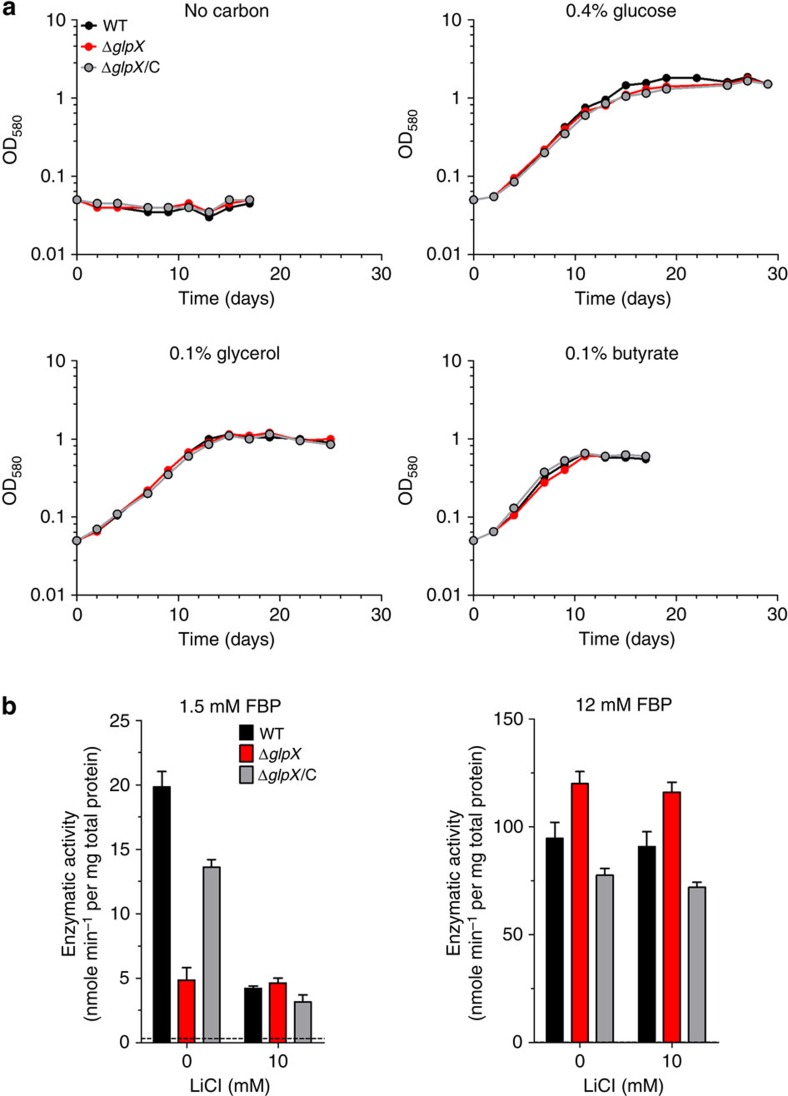
***Mtb*** **Δ*****glpX*** **grows on gluconeogenic carbon sources and has detectable FBPase activity.** (**a**) Growth of WT *Mtb* (black), Δ*glpX* (red) and complemented strain Δ*glpX*/C (grey) in Sauton’s minimal media containing no carbon source, 0.4% glucose, 0.1% glycerol or 0.1% butyrate. Data are representative of three independent experiments. (**b**) FBPase activity of WT *Mtb* (black), Δ*glpX* (red) and complemented strain Δ*glpX*/C (grey) cell lysates in the absence or presence of lithium chloride using 1.5 mM FBP or 12 mM FBP as substrate. Dashed line indicates limit of detection. Data are mean±s.d. of three biological replicates.

*GlpX* (*rv1099c*) is the only gene in the *Mtb* genome annotated to encode an FBPase. To study the role of FBPase in carbon metabolism, we generated Δ*glpX*, in which *glpX* was completely deleted and replaced with a hygromycin resistance cassette (Supplementary Fig. 1a). Southern blot analysis confirmed deletion of *glpX* (Supplementary Fig. 1b). Furthermore, GLPX protein was not detected from Δ*glpX* cell lysates by immunoblot and could be restored to levels observed in the wild-type (WT) strain by transformation of Δ*glpX* with a plasmid expressing *glpX* under the control of a constitutive promoter (Supplementary Fig. 1c). WT and Δ*glpX Mtb* strains grew indistinguishably with the glycolytic carbon source glucose (Fig. 2a). Contrary to our expectations, Δ*glpX* grew just as well as WT *Mtb* with gluconeogenic carbon sources such as glycerol, acetate and butyrate (Fig. 2a and Supplementary Fig. 6). These results suggest that *Mtb* remains gluconeogenically competent in the absence of the FBPase GLPX.

*Mtb* may express a second FBPase sustaining gluconeogenesis when GLPX is absent or utilize a metabolic bypass pathway that enables gluconeogenesis without the use of an FBPase. A metabolic bypass pathway is expected to result in changes in the Δ*glpX* metabolome that reflect the redirection of gluconeogenic carbon flow via such a putative bypass. Loss of *glpX*, however, led to no significant changes in the levels of metabolites that participate in gluconeogenesis/glycolysis (hexose-phosphate, pyruvate), the TCA cycle (α-ketoglutarate, oxaloacetate/aspartate) or the pentose phosphate pathway (sedoheptulose-phosphate) (Supplementary Fig. 2). While the lack of any significant changes in the Δ*glpX* metabolome does not support the existence of a metabolic bypass pathway, they demonstrate that gluconeogenesis in *Mtb* is not strictly dependent on GLPX. We thus hypothesized that *Mtb* may express a second FBPase that maintains gluconeogenesis in the absence of GLPX.

### Δ*glpX* retains detectable, lithium-resistant FBPase activity

To address whether *Mtb* expresses a second FBPase, we measured FBPase activity in lysates of Δ*glpX*. In the presence of a substrate concentration that is saturating for GLPX (1.5 mM FBP), we were able to detect FBPase activity from WT *Mtb* cell lysate (Fig. 2b). We also detected FBPase activity from Δ*glpX* cell lysate that was reduced by 76% relative to that of WT *Mtb* and could be complemented by expressing *glpX* in the Δ*glpX* background. The remaining FBPase activity in Δ*glpX* supports the hypothesis that *Mtb* has a redundant enzyme with FBPase activity. It has been previously reported that *Mtb’s* GLPX is sensitive to inhibition by lithium (IC_90 Li+_=2.5 mM)^23^. The mechanism of lithium inhibition is based on the ability of lithium ions to displace metal cofactors with a similar charge density like magnesium^21^,^27^, which is an important metal cofactor for *Mtb’s* GLPX^23^. In the presence of 10 mM LiCl, FBPase activity in WT was reduced to levels that were similar to those observed in Δ*glpX*. In contrast, FBPase activity in Δ*glpX* was not inhibited by the same LiCl treatment. Thus, WT *Mtb* FBPase activity is partially sensitive to lithium, and the lithium-resistant portion was retained in Δ*glpX*. Unlike GLPX, *Mtb*’s second FBPase appears to be lithium resistant. In the presence of a higher substrate concentration (12 mM FBP), we observed increased FBPase activity levels in both WT and Δ*glpX*, and there was no significant difference in the FBPase activity levels of these strains (Fig. 2b). Furthermore, lithium did not inhibit FBPase activity of these strains at this higher substrate concentration. The fact that FBPase activity in Δ*glpX* increased with increasing substrate concentration suggests that *Mtb*’s second FBPase has a lower affinity (that is, higher *K*_m_) for FBP than GLPX. The residual FBPase activity detected in Δ*glpX* lysates could be inactivated by heating the lysate to ≥80 °C (data not shown). Taken together, these data support the existence of a second FBPase in *Mtb* that differs from GLPX in at least its substrate affinity and resistance to lithium inhibition.

### Identification of the second FBPase in *Mtb*

**Figure 3:**
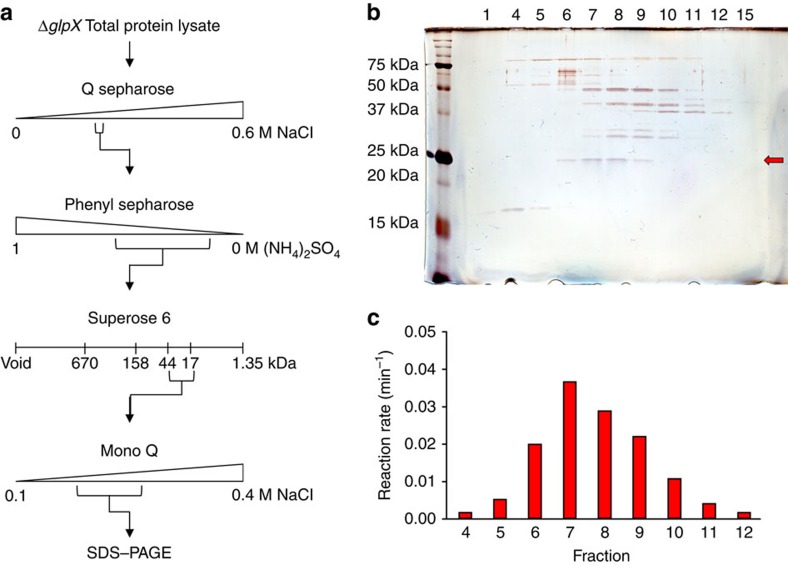
Identification of the second FBPase by biochemical extraction. (**a**) FBPase activity of the Δ*glpX* cell lysate was purified using a series of liquid chromatography techniques followed by SDS–PAGE of the final active fractions. (**b**,**c**) Silver-stained SDS–PAGE gel (**b**) and FBPase activity profile using 12 mM FBP as substrate (**c**) for the range of fractions from the final Mono Q anion exchange chromatography with detectable FBPase activity. Inactive fractions 1 and 15 were included in the SDS–PAGE analysis. A single band of ∼25 kDa (red arrow in **b**) correlated with the FBPase activity profile of the fractions. Peptide mass fingerprinting identified the major protein component of this band to be GPM2 (Rv3214, molecular weight=21.95 kDa, 66.5% coverage, eight peptides).

To identify *Mtb*’s second FBPase, we took an unbiased biochemical approach and purified the FBPase activity from Δ*glpX* lysate using a series of chromatographic enrichments (Fig. 3a). The purification scheme involved four steps that separate proteins based on their charge, hydrophobicity and molecular weight. After each purification step, active fractions were identified using an FBPase activity assay and then pooled for further purification. Consistent with our earlier observations, active fractions demonstrated robust FBPase activity in the presence of 12 mM FBP and were resistant to inhibition by lithium (tested in the presence of 1.5 mM FBP). After four purification steps, FBPase activity was enriched by at least 65-fold (Supplementary Table 1). We then visualized the remaining proteins in the final active fractions by SDS–polyacrylamide gel electrophoresis (SDS–PAGE; Fig. 3b). We observed a single band of ∼25 kDa whose intensity correlated with the FBPase activity profile of the active fractions (Fig. 3c). Peptide mass fingerprinting identified the protein in this band as GPM2 (Rv3214, molecular weight=21.95 kDa, 66.5% coverage, eight peptides, Supplementary Table 2), which has been reported to function as an acid phosphatase^26^.

GPM2 can dephosphorylate a variety of substrates including phosphorylated sugars like F6P^26^. FBP, however, had not been tested. While GPM2 had not been tested for sensitivity to lithium inhibition, it does not appear to require a metal cofactor, suggesting that it might be lithium resistant. While GPM2 does not belong to any of the five types of FBPases, it shares 31% identity with *Saccharomyces cerevisiae* YK23 (also known as SHB17), which has both FBPase^28^ and sedoheptulose bisphosphatase (SBPase) activity^29^. YK23 has a relatively high *K*_m_ for FBP (0.5 mM) and also lacks a metal cofactor^28^. Based on this evidence, we hypothesized that GPM2 may serve as an FBPase in *Mtb*.

### Validation of GPM2 as an FBPase

**Figure 4:**
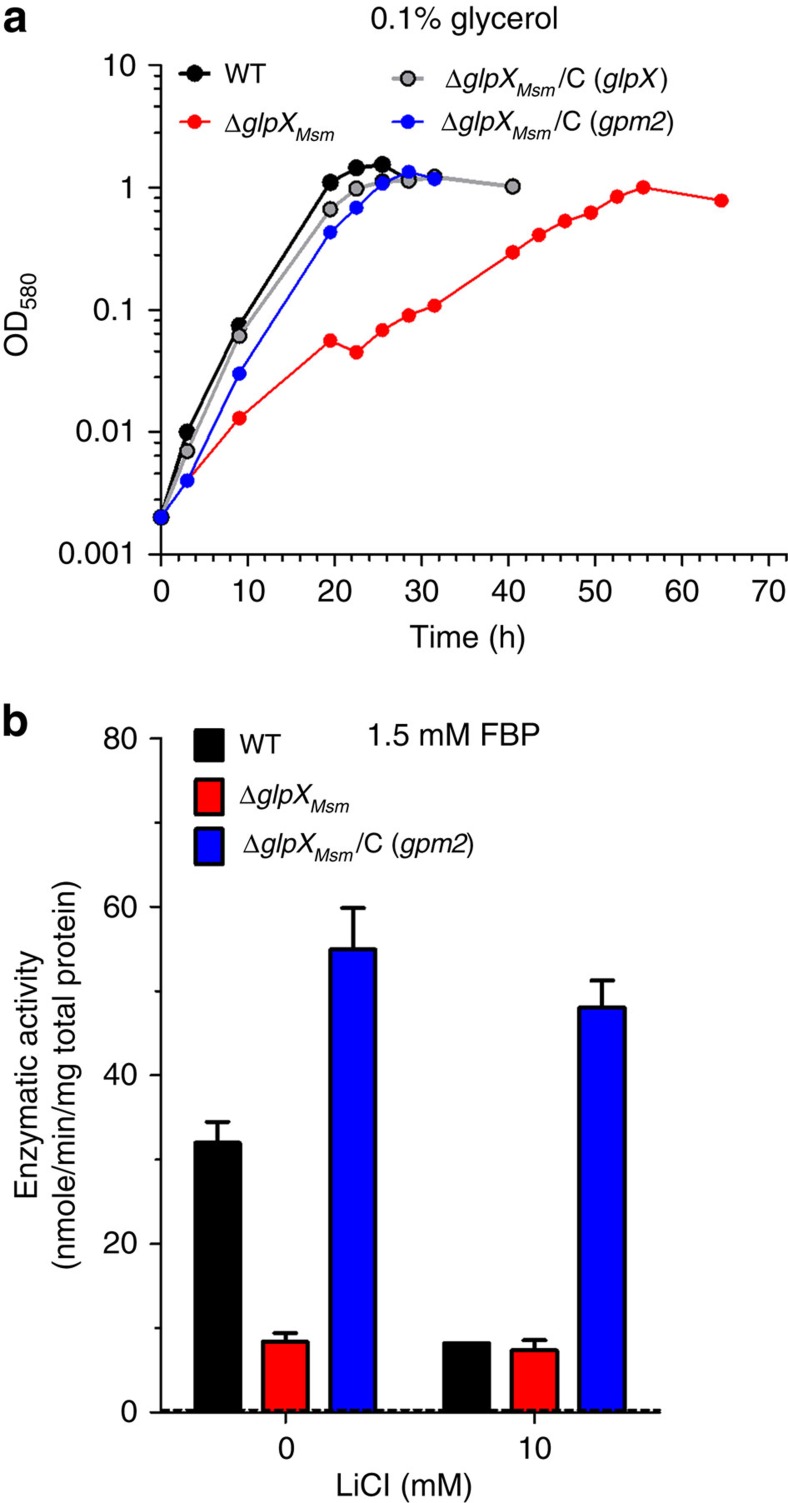
***Gpm2*** **complements Δ*****glpX***_***Msm***_ **growth and FBPase activity defects.** (**a**) Growth of WT *Msm* (black), Δ*glpX*_*Msm*_ (red) and complemented strains Δ*glpX*_*Msm*_/C (*glpX*) (grey) or Δ*glpX*_*Msm*_/C (*gpm2*) (blue) in 7H9 media with 0.1% glycerol as the sole carbon source. Data are representative of two independent experiments. (**b**) FBPase activity of WT *Msm* (black), Δ*glpX*_*Msm*_ (red) and complemented strain Δ*glpX*_*Msm*_/C (*gpm2*) (blue) cell lysates in the absence or presence of lithium chloride using 1.5 mM FBP as substrate. Dashed line indicates limit of detection. Data are mean±s.d. of three biological replicates.

Expression of GLPX in an FBPase-deficient *E. coli* strain restored its ability to grow on gluconeogenic carbon sources^22^. We sought to perform a similar functional complementation test with GPM2 to validate it as an FBPase. We generated a *Mycobacterium smegmatis* (*Msm*) strain with a deletion of the *glpX* homologue, Δ*glpX*_*Msm*_ (Supplementary Fig. 3). While Δ*glpX*_*Msm*_ grew well on glucose, this strain demonstrated a partial growth defect on gluconeogenic carbon sources (Supplementary Fig. 4a). Similar to what was observed in *Mtb*, Δ*glpX*_*Msm*_ had reduced FBPase activity relative to WT and the residual activity was resistant to inhibition by lithium (Supplementary Fig. 4b). The partial growth defect of Δ*glpX*_*Msm*_ on gluconeogenic carbon sources could be fully complemented by overexpressing GLPX from *Mtb* (Supplementary Figs 4a and 3c). Overexpression of GLPX in Δ*glpX*_*Msm*_ also complemented the FBPase activity defect (Supplementary Fig. 4b). These results demonstrate that expression of an FBPase can functionally complement the growth and FBPase activity defects of Δ*glpX*_*Msm*_. We reasoned that Δ*glpX*_*Msm*_ could be similarly used to test whether GPM2 can function as an FBPase *in vivo*. Indeed, overexpression of GPM2 in Δ*glpX*_*Msm*_ complemented the mutant’s partial growth defect on glycerol just as well as GLPX overexpression (Fig. 4a) and complemented the FBPase activity defect of Δ*glpX*_*Msm*_ (Fig. 4b). Moreover, the additional FBPase activity provided by GPM2 overexpression was resistant to inhibition by lithium.

**Figure 5:**
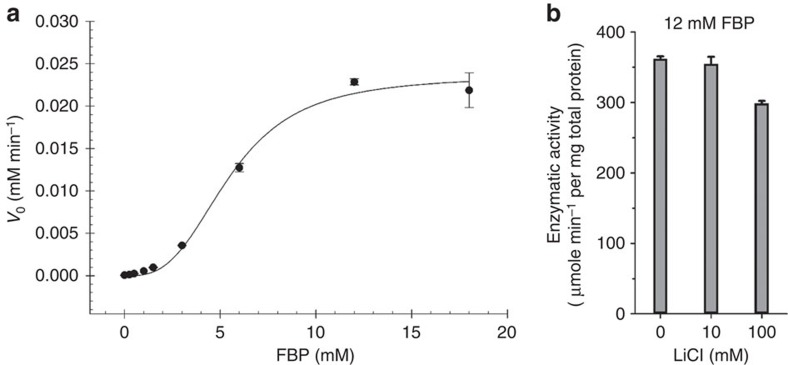
GPM2 has lithium-resistant FBPase activity. (**a**) FBPase activity progress curve for recombinant His-tagged GPM2. Data are mean±s.d. of three technical replicates. Plotted line represents the non-linear fit for the three parameter Hill equation (*r*^2^=0.99073255, s.e. of estimate=0.0009). (**b**) Recombinant GPM2 FBPase activity in the presence of 12 mM FBP and varying amounts of lithium chloride. Data are mean±s.d. of three technical replicates.

To further validate GPM2 as an FBPase, we determined GPM2’s kinetic parameters with FBP as a substrate. Recombinant GPM2 demonstrated robust FBPase activity (Fig. 5a and Table 1). The enzyme’s FBPase activity followed allosteric sigmoidal kinetics with both a high *K*_m_ (5.51 mM FBP) and a high *k*_cat_ (1.87 × 10^2^ s^−1^) compared with GLPX. GPM2 FBPase activity was also resistant to inhibition by lithium (Fig. 5b). Thus, the enzymatic characteristics of GPM2 match the properties of the remaining FBPase activity observed in Δ*glpX* cell lysates. Both GLPX and GPM2 have specificity constants (*k*_cat_/*K*_m_) in the same range (2.3 × 10^4^ s^−1^ M^−1^ and 3.4 × 10^4^ s^−1^ M^−1^, respectively), suggesting that they function equally well as FBPases.

**Figure 6:**
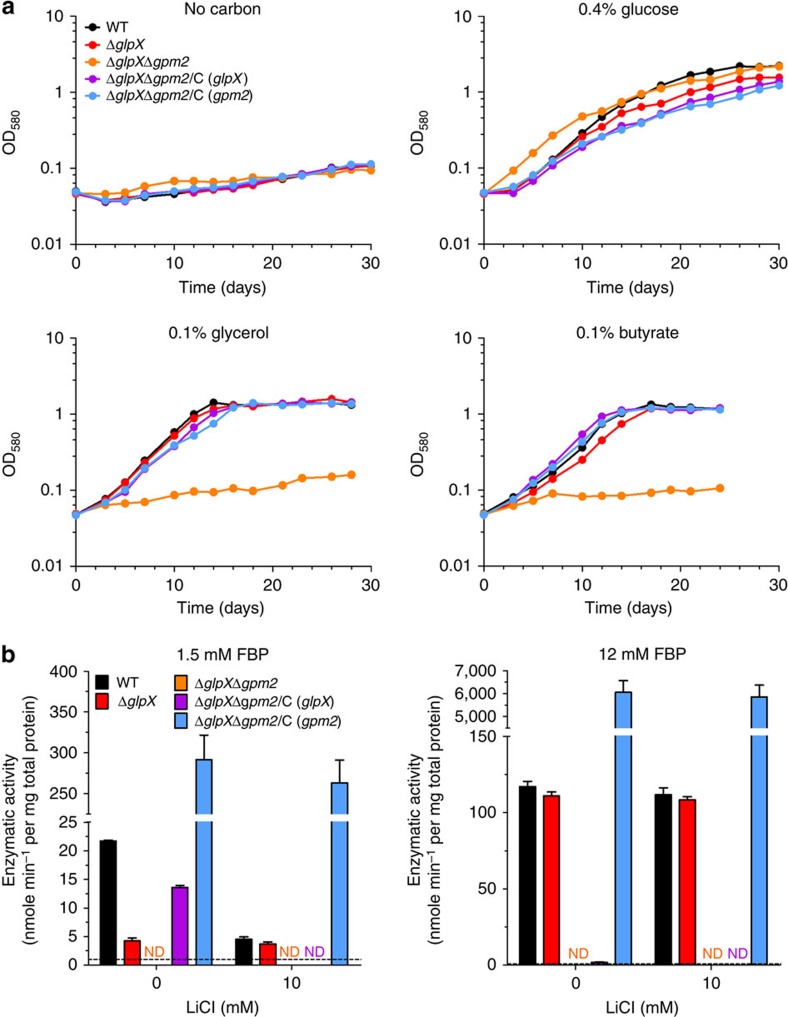
***Mtb*** **Δ*****glpX*****Δg*****pm2*** **fails to grow on gluconeogenic carbon sources and lacks detectable FBPase activity.** (**a**) Growth of WT *Mtb* (black), Δ*glpX* (red), Δ*glpX*Δg*pm2* (orange) and complemented strains Δ*glpX*Δg*pm2*/C (*glpX*) (purple) and Δ*glpX*Δg*pm2*/C (*gpm2*) (blue) in Sauton’s minimal media containing no carbon source, 0.4% glucose, 0.1% glycerol or 0.1% butyrate. Data are representative of three independent experiments. (**b**) FBPase activity of WT *Mtb* (black), Δ*glpX* (red), Δ*glpX*Δg*pm2* (orange) and complemented strains Δ*glpX*Δg*pm2*/C (*glpX*) (purple) and Δ*glpX*Δg*pm2*/C (*gpm2*) (blue) cell lysates in the absence or presence of lithium chloride using 1.5 mM FBP or 12 mM FBP as substrate. ND indicates no detectable FBPase activity. Dashed line indicates limit of detection. Data are mean±s.d. of three biological replicates.

Was GPM2 responsible for the ability of Δ*glpX* to grow on gluconeogenic carbon sources? To address this question, we deleted *gpm2* in the Δ*glpX Mtb* mutant background (Supplementary Fig. 5). While Δ*glpX*Δ*gpm2* grew as well as WT on glucose, it was unable to grow on any of the gluconeogenic carbon sources that we tested: glycerol, acetate and butyrate (Fig. 6a and Supplementary Fig. 6). Thus, the ability of Δ*glpX* to grow on gluconeogenic substrates was dependent on the expression of GPM2. The growth defect of Δ*glpX*Δ*gpm2* on gluconeogenic carbon sources was fully complemented by restoring expression of either GLPX or GPM2 (Fig. 6a and Supplementary Figs 5c,d and 6), demonstrating that *Mtb* requires expression of at least one enzyme with FBPase activity for gluconeogenic growth. Moreover, Δ*glpX*Δ*gpm2* lacked detectable FBPase activity at any substrate concentration that we tested (Fig. 6b), suggesting that the strain’s inability to grow on gluconeogenic substrates is due to disruption of gluconeogenesis at the FBPase reaction step. Consistent with this, FBPase activity was restored in Δ*glpX*Δ*gpm2* expressing either GLPX or GPM2 (Fig. 6b). The extent of complementation of FBPase activity with GLPX or GPM2 reflected the properties of each enzyme. Expression of GLPX in Δ*glpX*Δ*gpm2* provided lithium-sensitive FBPase activity that was only observed with 1.5 mM FBP, but not detected with 12 mM FBP, consistent with substrate inhibition of GLPX at high substrate concentrations^23^. In contrast, expression of GPM2 in Δ*glpX*Δ*gpm2* generated lithium-resistant FBPase activity, and, consistent with the higher *K*_m_ of GPM2, this activity increased with higher FBP concentrations.

### Gluconeogenesis is disrupted in Δ*glpX*Δ*gpm2*

**Figure 7:**
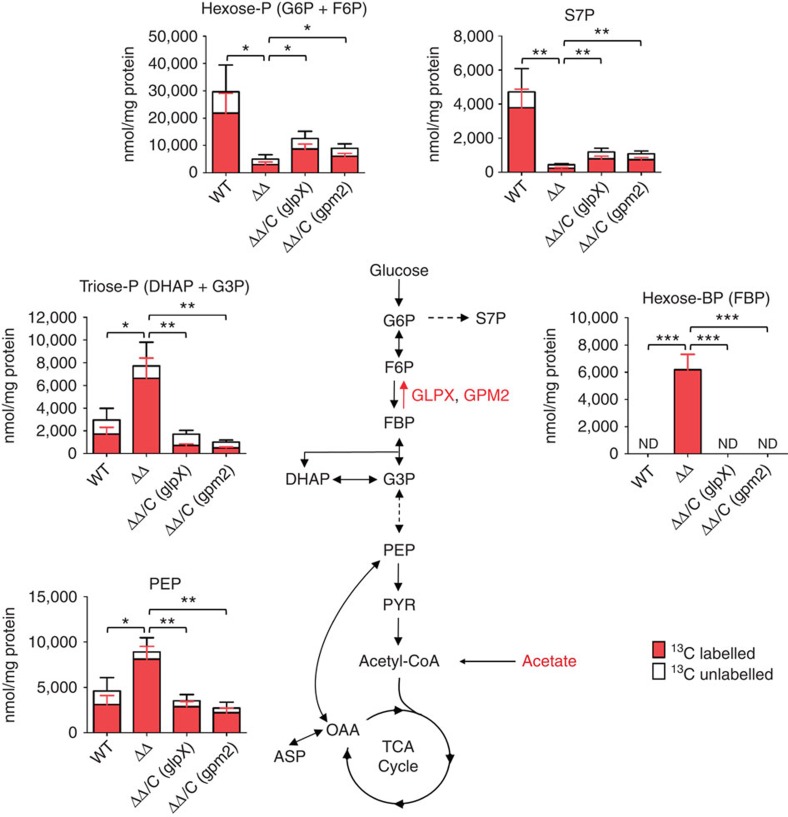
**Gluconeogenesis is disrupted in** ***Mtb*** **Δ*****glpX*****Δg*****pm2***. Abundance and ^13^C labelling of metabolites in WT *Mtb*, Δ*glpX*Δg*pm2* (ΔΔ) and complemented strains Δ*glpX*Δg*pm2*/C (*glpX*) (ΔΔ/C (glpX)) and Δ*glpX*Δg*pm2*/C (*gpm2*) (ΔΔ/C (gpm2)). Bacteria were grown on glucose-containing plates for 5 days and then transferred to U-^13^C acetate-containing plates for 24 h prior to harvesting. Data are mean±s.d. of three biological replicates and are representative of two independent experiments. 0.01<*P≤0.05, 0.001<**P≤0.01, ***P≤0.001 by Student’s *t*-test. ND indicates that FBP was below the limit of detection (3.13 μM). ASP, aspartate; DHAP, dihydroxyacetone phosphate; F6P, fructose 6-phosphate; FBP, fructose 1,6-bisphosphate; G3P, glyceraldehyde 3-phosphate; G6P, glucose 6-phosphate; OAA, oxaloacetate; PEP, phosphoenolpyruvate; PYR, pyruvate; S7P, sedoheptulose 7-phosphate.

We next examined the metabolic state of Δ*glpX*Δ*gpm2 Mtb* in the presence of a gluconeogenic carbon source using a filter-based metabolomics platform^30^. Bacteria were first grown on 0.2% glucose for 5 days to obtain sufficient biomass and then transferred to a universally ^13^C-labelled (U-^13^C) carbon source (0.2% acetate or 0.2% glucose) for 24 h. When Δ*glpX*Δ*gpm2* was switched from glucose to U-^13^C-labelled acetate (Fig. 7), phosphoenoylpyruvate (PEP) and the triose-phosphates (triose-P)—metabolites upstream of the FBPase-catalysed reaction in gluconeogenesis—accumulated. While FBP levels recovered from WT *Mtb* were below our limit of detection, we detected FBP in Δ*glpX*Δ*gpm2*, suggesting accumulation of the FBPase substrate in this mutant. Δ*glpX*Δ*gpm2* also had decreased pool sizes of downstream metabolites like the hexose phosphates (hexose-P), which include the FBPase product F6P, and the pentose phosphate pathway intermediate sedoheptulose 7-phosphate (S7P). Incorporation of ^13^C from U-^13^C acetate into these downstream metabolites was decreased as well. These results indicate a disruption of gluconeogenesis at the FBPase-catalysed reaction, which is consistent with the loss of both FBPases in Δ*glpX*Δ*gpm2* and the lack of FBPase activity. This metabolic defect was relieved by expression of either GLPX or GPM2, which reversed to a large degree both the changes in pool size of upstream and downstream metabolites and ^13^C labelling of hexose-P and S7P. Thus, expression of either of the two enzymes with FBPase activity is sufficient for functional gluconeogenesis in *Mtb*. Notably, the restoration of gluconeogenic carbon flux through the FBPase reaction step upon expression of GPM2 supports this enzyme’s activity as an FBPase *in vivo*.

When Δ*glpX*Δ*gpm2* was grown on U-^13^C glucose (Supplementary Fig. 7), there were no significant changes in pool sizes or ^13^C labelling of metabolites in central carbon metabolism, indicating that glycolysis was not disrupted in Δ*glpX*Δ*gpm2*. This is consistent with the ability of Δ*glpX*Δ*gpm2* to grow on glucose. Collectively, our metabolomic analyses suggest that loss of FBPase activity in Δ*glpX*Δ*gpm2* results in a specific disruption of gluconeogenesis at the FBPase-catalysed reaction, rendering *Mtb* unable to grow on gluconeogenic substrates.

Deletion mutants of some enzymes in the gluconeogenesis pathway, such as FBA and TPI, were not only unable to grow using fatty acids but died in the presence of this carbon source^9^,^10^. We found that Δ*glpX*Δ*gpm2* remained viable in media containing 0.2% acetate for at least 28 days (Supplementary Fig. 6b). Fatty acids were not toxic to Δ*glpX*Δ*gpm2* when provided in combination with glucose (Supplementary Fig. 8). On the contrary, Δ*glpX*Δ*gpm2* grew more robustly with 0.2% glucose and 0.1% acetate than with 0.4% glucose alone.

### FBPase activity is required for *Mtb* virulence

**Figure 8:**
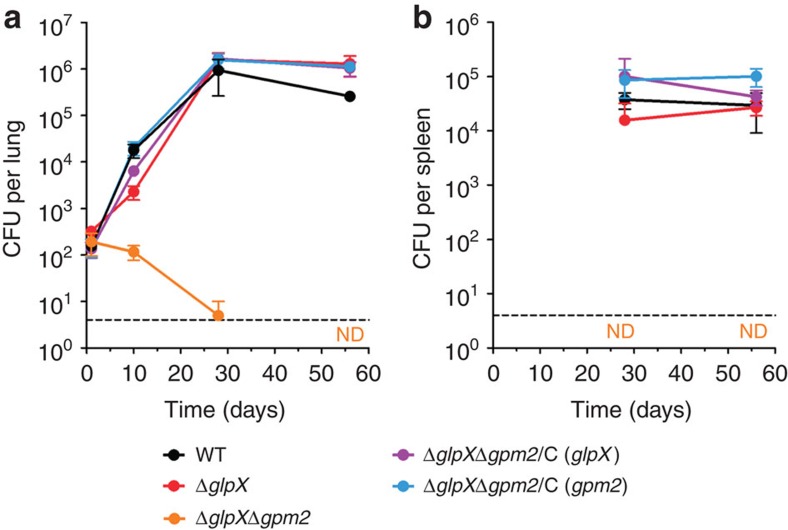
***Mtb*** **Δ*****glpX*****Δg*****pm2*** **is attenuated** ***in vivo***. (**a**) Lung c.f.u. from C57BL/6 mice infected with WT *Mtb* (black), Δ*glpX* (red), Δ*glpX*Δg*pm2* (orange) and complemented strains Δ*glpX*Δg*pm2*/C (*glpX*) (purple) and Δ*glpX*Δg*pm2*/C (*gpm2*) (blue). ND indicates no detectable c.f.u. burden for Δ*glpX*Δg*pm2* at day 56. Data represent the mean±s.d. of four mice per time point. Dashed line indicates limit of detection of 4 c.f.u. per lung. (**b**) Spleen c.f.u. from C57BL/6 mice infected with WT *Mtb* (black), Δ*glpX* (red), Δ*glpX*Δg*pm2* (orange) and complemented strains Δ*glpX*Δg*pm2*/C (*glpX*) (purple) and Δ*glpX*Δg*pm2*/C (*gpm2*) (blue). ND indicates no detectable c.f.u. burden for Δ*glpX*Δg*pm2* at day 28 and day 56. Data represent the mean±s.d. of four mice per time point. Dashed line indicates limit of detection of 4 c.f.u. per spleen.

We next evaluated whether FBPase activity and gluconeogenesis are required during an *Mtb* infection. In a mouse model of *Mtb* infection, Δ*glpX* was able to replicate and persist in lungs similar to the WT strain (Fig. 8a). In contrast, Δ*glpX*Δ*gpm2* failed to replicate in mouse lungs during the first 10 days of infection and began to die thereafter. Δ*glpX*Δ*gpm2* was effectively cleared from the host by 56 days post infection when the strain’s c.f.u. burden was below the limit of detection (4 c.f.u. per lung). While WT *Mtb* and Δ*glpX* were detected at comparable levels in mouse spleens at both 28 and 56 days post infection, we were unable to detect Δ*glpX*Δ*gpm2* at either time point (Fig. 8b). The clearance of Δ*glpX*Δ*gpm2* from mouse lungs was also evident from lung histopathology. Only mice infected with Δ*glpX*Δ*gpm2* lacked visible lung lesions at 56 days post infection (Supplementary Fig. 9). The severe attenuation of Δ*glpX*Δ*gpm2 in vivo* indicates that both FBPase activity and gluconeogenesis are required for *Mtb* virulence. The attenuation of Δ*glpX*Δ*gpm2* in mouse lungs and spleens could be fully complemented by expressing either GLPX or GPM2 in the Δ*glpX*Δ*gpm2* background. Thus, *Mtb* requires expression of at least one enzyme with FBPase activity to establish infection.

## Discussion

Our work demonstrates that, in addition to GLPX, *Mtb* expresses a second FBPase, GPM2. The existence of a second FBPase in *Mtb* has been suggested before by transposon mutagenesis studies^24^,^25^. The inositol monophosphate phosphatase (IMPase) CYSQ (Rv2131c) was put forth as a possible second FBPase because the recombinant enzyme has FBPase activity *in vitro*^31^. However, CYSQ has since been shown to function in sulfur metabolism as a 3′-phosphoadenosine-5′-phosphatase (PAPase)^32^. Not only does CYSQ have a higher *k*_cat_/*K*_m_ with PAP as substrate than with IMP or FBP, but expression of CYSQ complemented the sulfite auxotrophy of *E. coli* Δ*cysQ*, demonstrating that this enzyme functions as a PAPase *in vivo*. Along with the other IMPase homologues—SUHB, IMPA and IMPC—CYSQ contains a sequence motif, which defines a phosphomonoesterase superfamily that includes lithium-sensitive IMPases and FBPases^21^. While we initially considered the IMPase homologues as candidates for the second FBPase, our finding that Δ*glpX* FBPase activity was lithium resistant ruled out these candidates. There are species with both a Type I and a Type II FBPase (*E. coli*)^12^ or a Type II and Type III FBPase (*Bacillus subtilis*)^33^, but mycobacteria lack Type I and Type III FBPase homologues. Thus, the second FBPase activity likely derived from a non-classical FBPase. Proof-of-concept for an unrelated phosphatase supporting FBPase activity in the absence of a classical FBPase was previously demonstrated in *E. coli*, where expression of an alkaline phosphatase complemented the growth defect of Δ*fbp* on glycerol^34^.

Our observations of gluconeogenic growth and FBPase activity in an FBPase mutant are similar to those reported for the yeast *Yarrowia lipolytica*^35^. Deletion of the Type I FBPase in *Y. lipolytica* resulted in a partial growth defect on gluconeogenic carbon sources. Preliminary enzymology demonstrated that this mutant had detectable, lithium-resistant FBPase activity due to an ‘alternative phosphatase’ with a high *K*_m_ for FBP. The second FBPase in this species remains to be identified. Nonetheless, it is apparent that functional redundancy between a classical FBPase and an alternative phosphatase can occur in other organisms. Our observation of similar results in *Msm* is consistent with the existence of a GPM2 homologue in this species: MSMEG_1926 (69% identity with GPM2). Thus, FBPase redundancy may extend to other mycobacterial species with GPM2 homologues.

The predicted function of GPM2 has changed over time. Initially, the protein was annotated based on homology to the *E. coli* 4’-phosphopantetheinyl transferase ENTD, which is involved in siderophore biosynthesis. An alternative classification placed the enzyme as a member of the cofactor-dependent phosphoglycerate mutase (dPGM) family^36^ from which the current GPM2 name is derived. Phosphoglycerate mutases operate in the gluconeogenesis pathway, driving the conversion of 2-phosphoglycerate to 3-phosphoglycerate via a 2,3-bisphosphoglycerate intermediate. Functional assays and structural analysis by Watkins and Baker^26^ showed that GPM2 more closely resembles members of the broad-specificity phosphatase subfamily within the dPGM family. The authors determined that GPM2 had negligible mutase activity and that the enzyme’s function was more accurately described as a broad-spectrum acid phosphatase^26^. Because of GPM2’s ability to dephosphorylate a variety of substrates, it has been suggested that GPM2 serves as a phosphate scavenging enzyme, enabling *Mtb* to obtain phosphate from host molecules or its own metabolites inside the nutrient-limiting environment of the macrophage^26^. Indeed, *gpm2* expression was upregulated during phosphate starvation^37^, suggesting that GPM2 might play a role during phosphate starvation. Our work describes a previously unknown function of GPM2. While GPM2 was once hypothesized to play a role in gluconeogenesis as a phosphoglycerate mutase, we now demonstrate that this enzyme maintains this pathway as an FBPase when GLPX is absent. This new function is consistent with its previously reported ability to act on phosphorylated sugars. Our findings neither support nor rule out GPM2’s role in maintaining the mycobacterial phosphate pool, but show that this enzyme can serve a redundant role to a more specialized enzyme. Based on our work and that of Watkins *et al.*^26^, we propose that the function of GPM2 as a sugar phosphatase be recognized in the future with a new name for this enzyme: SUP1. Since gluconeogenesis is critical to *Mtb*’s ability to establish infection, the conservation of GPM2 is likely a byproduct of the high selective pressure on *Mtb* to maintain a functional gluconeogenic pathway. Given our results, GPM2 could potentially compensate for the loss of other specialized phosphatases in other contexts, providing redundancy to other *Mtb* metabolic pathways that are critical to virulence.

Our enzymology reveals that GPM2 has different properties from those of the classical FBPase GLPX. With its lower *K*_m_, GLPX has a higher affinity for FBP with optimal FBPase activity in the mid-micromolar substrate concentration range. The higher *K*_m_ of GPM2, on the other hand, confers optimal FBPase activity in the millimolar range. Although GPM2 has a lower affinity for FBP, the enzyme’s higher *k*_cat_ makes it a robust FBPase when supplied with enough substrate. The different kinetics of GLPX and GPM2 suggest that these enzymes likely play different roles as FBPases. GPM2’s allosteric sigmoidal kinetics suggests that this enzyme’s robust FBPase activity at high FBP concentrations is substrate-tunable. In contrast, GLPX’s Michaelis–Menten kinetics make this enzyme less sensitive to substrate concentration, resulting in a core low level activity at lower FBP concentrations. While GPM2 can provide sufficient FBPase activity in the absence of GLPX, it is not entirely clear how this occurs. Since the two enzymes have different substrate affinities, GLPX and GPM2 are not active as FBPases at the same substrate concentration. Under WT *Mtb* conditions, FBP levels are likely optimized for GLPX over GPM2 FBPase activity. Loss of GLPX in *Mtb*, however, leads to a buildup of FBP that allows GPM2 FBPase activity, and effectively maintains gluconeogenesis in the absence of the classical FBPase. Such substrate-level control of enzymatic reactions has been reported before. In *E. coli*, a transaldolase deletion mutant can unexpectedly grow on xylose due to an accumulation of S7P that supports alternative 7-phosphosedohepturo-kinase activity from the 6-phosphofructokinase PFKA, driving a functional bypass of the blocked transaldolase reaction step^38^. The FBP concentration in *Mtb* is undetermined. In *E. coli*, levels of FBP can range from 15 mM when cultured on glucose to <150 μM when cultured on acetate^39^. The FBP concentration in *B. subtilis* was 4.3 mM when grown on glycerol as the sole carbon source^40^. Millimolar FBP concentrations similar to those observed in *E. coli* and *B. subtilis* would be sufficient to support GPM2 FBPase activity.

We sought to address whether gluconeogenesis is required for *Mtb* virulence. By deleting both *glpX* and *gpm2* in *Mtb*, we generated a strain that lacks detectable FBPase activity and, as a consequence, suffers from defective gluconeogenesis as evidenced from *in vitro* growth assays and metabolomics analysis. The attenuation of Δ*glpX*Δ*gpm2* in the mouse model indicates that *Mtb* requires gluconeogenesis for virulence. The bacterial burden of Δ*glpX*Δ*gpm2* was significantly lower than that of WT as early as 10 days post infection, suggesting that *Mtb* requires gluconeogenesis early during the course of an infection. Glycolysis, which is intact in Δ*glpX*Δ*gpm2*, was not sufficient to support replication of Δ*glpX*Δ*gpm2 in vivo*. These results are consistent with an earlier study that showed that glycolysis was dispensable for establishing infection but played a role in persistence^41^. We also observed killing and eventual clearance of Δ*glpX*Δ*gpm2* in mouse lungs, indicating that gluconeogenesis is necessary for survival of *Mtb* in the host. The mechanism resulting in death of Δ*glpX*Δ*gpm2 in vivo* awaits future study. Disruption of gluconeogenesis at the FBPase reaction step in Δ*glpX*Δ*gpm2* might kill the bacterium through the accumulation of a toxic upstream metabolite. For instance, accumulation of FBP could be a source of toxicity, as was suggested for an *E. coli* FBA deletion mutant^42^,^43^. However, while Δ*glpX*Δ*gpm2* accumulates FBP and other metabolites upstream of the FBPase reaction when grown on acetate *in vitro*, this strain does not die in this condition. On the contrary, growth of Δ*glpX*Δ*gpm2* on glucose was enhanced by acetate, suggesting that *Mtb* may still be able to co-catabolize these substrates^30^ in the absence of FBPase activity. Therefore, *in vivo* killing of Δ*glpX*Δ*gpm2* is likely not due to accumulation of phosphorylated metabolites. The discrepancy between the *in vitro* and *in vivo* survival of Δ*glpX*Δ*gpm2* mirrors the results obtained with Δ*pckA*, another mutant with a gluconeogenesis-specific defect^7^. Loss of gluconeogenesis may contribute to the *in vivo* attenuation of both strains by producing a common set of metabolic changes that predispose the bacteria to killing via anti-microbial mechanisms in the host.

GLPX has been considered as a drug target for TB chemotherapy^23^. Humans lack a Type II FBPase, allowing design of a species-specific drug that would selectively target *Mtb* gluconeogenesis^44^. While our study highlights *Mtb* gluconeogenesis as critical to the pathogen’s ability to survive in the host, it also suggests that blocking this pathway at the FBPase step would be challenging. A drug targeting GLPX would not be sufficient to inhibit growth or kill *Mtb*, as the pathogen can utilize GPM2 to maintain gluconeogenic carbon flux. Furthermore, GLPX and GPM2 belong to different enzyme families. FBPase activity of the phosphomonoesterase family member GLPX involves metal cofactors coordinating and activating a water nucleophile to attack the phosphorus 1 atom^45^. In contrast, the phosphatase activity of the dPGM family member GPM2 does not involve a metal cofactor and uses a phosphohistidine catalytic intermediate instead^46^. These differences in the catalytic mechanisms of GLPX and GPM2 FBPases will hamper design of a single inhibitor against both enzymes.

In conclusion, we report previously undescribed redundancy in FBPase activity in *Mtb* that complicates the design of FBPase-targeting drugs. We also demonstrate the importance of gluconeogenesis for *Mtb* virulence. A better understanding of how defective gluconeogenesis contributes to clearance of *Mtb in vivo* could identify alternative targets for new TB drugs.

## Methods

### Strains and culture conditions

*Mtb* H37Rv (obtained from the Trudeau Institute) was cultured in Middlebrook 7H9 containing 0.05% Tween-80, 0.5% bovine serum albumin fraction V, 0.2% glucose, 0.085% NaCl, 0.2% glycerol and incubated standing at 37 °C with 5% CO_2_. For Δ*glpX*Δ*gpm2*, Middlebrook 7H9 containing 0.05% Tween-80, 0.5% bovine serum albumin fraction V, 0.4% glucose and 0.085% NaCl was used. For solid agar, Middlebrook 7H10 agar with 0.5% glycerol and 10% OADC enrichment (0.5% bovine serum albumin fraction V, 0.2% glucose, 0.085% NaCl, 0.006% oleic acid and 0.0003% catalase at final concentration) was used. For Δ*glpX*Δ*gpm2*, Middlebrook 7H10 agar with 0.4% glucose, 0.5% bovine serum albumin fraction V and 0.085% NaCl was used. *Mtb* growth curves were performed using carbon-defined Sauton’s minimal media containing 0.05% potassium phosphate monobasic, 0.05% magnesium sulfate heptahydrate, 0.2% citric acid, 0.005% ferric ammonium citrate, 0.05% ammonium sulfate, 0.0001% zinc sulfate and 0.05% Tyloxapol adjusted to pH 7.4. For enzyme activity assays, strains were cultured in Middlebrook 7H9 containing 0.05% Tween-80, 0.5% bovine serum albumin fraction V, 0.085% NaCl and either 0.2% glycerol or 0.4% glucose as the sole carbon source. For metabolomics analysis of Δ*glpX*Δ*gpm2*, *Mtb* was seeded onto 0.22 μm nitrocellulose filters at OD_580_=1, 1 ml per filter. Filters were placed on Middlebrook 7H10 agar with 0.2% glucose, 0.5% bovine serum albumin fraction V, and 0.085% NaCl and incubated at 37 °C with 5% CO_2_ for 5 days. Filters were then transferred to plates of similar composition with different carbon sources (0.2% glucose, 0.2% U-^13^C glucose, 0.2% acetate or 0.2% U-^13^C acetate) and incubated for 24 h before harvesting by quenching in acetonitrile/methanol/dH_2_O (40:40:20) on dry ice followed by mechanical lysis by bead beating with 0.1 mm zirconia/silica beads and clarification using a 0.22 μm filter as described before^47^. For metabolomics analysis of Δ*glpX*, filters were placed on Middlebrook 7H10 agar with 0.2% glycerol, 0.5% bovine serum albumin fraction V, and 0.085% NaCl and incubated at 37 °C with 5% CO_2_ for 5 days. Filters were then transferred to plates with either 0.2% glycerol or 0.2% U-^13^C glycerol and incubated for 16 h before harvesting.

*Msm* mc^2^155 (ATCC 700084) was cultured in Middlebrook 7H9 containing 0.05% Tween-80, 0.2% glycerol, 0.4% glucose, and incubated at 37 °C with 5% CO_2_ and light shaking. The same media composition was used when culturing for enzyme activity assays. For solid media, Middlebrook 7H10 agar with 0.2% glycerol and 0.4% glucose was used. Growth curves were performed with carbon-defined Middlebrook 7H9 containing 0.05% Tyloxapol and adjusted to pH 7.0.

Carbon sources were provided at the described concentration as w/v or v/v. 50 μg ml^−1^ hygromycin B, 25 μg ml^−1^ kanamycin and 25 μg ml^−1^ zeocin were used for selection where appropriate.

### Mutant generation and validation

All vectors used for mutant generation and complementation were constructed using Gateway Cloning Technology (Invitrogen). Primer sequences are provided in Supplementary Table 3. Mutant genotypes were confirmed by PCR and Southern blot analysis.

Δ*glpX* was generated using allelic exchange and specialized transducing phage phAE87 (ref. 48). Approximately 500 bp fragments containing the upstream and downstream region of the *glpX* gene were amplified by PCR and cloned into pJSC284-loxP flanking the hygromycin resistance gene. We generated pJSC284-loxP, a derivative of pJSC284 (gift from Jeff S. Cox), that contains loxP sites flanking the hygromycin cassette. The plasmid was digested with PacI and packaged into the unique PacI site of the temperature-sensitive phage phAE87. The phage was amplified in *Msm* at 30 °C and used to infect *Mtb* as described previously^49^. For complementation of Δ*glpX*, we generated pGMCK-pTb21-glpX, a vector that expresses *glpX* driven by the constitutive Tb21 promoter and integrates into the attL5 site of the *Mtb* genome.

Δ*glpX*_*Msm*_ was generated using a suicide plasmid approach. Approximately 800 bp fragments corresponding to regions upstream and downstream of *msmeg_5239* (*glpX* homologue) were amplified by PCR and cloned into a temperature-sensitive vector pDE43-XSTS to flank the hygromycin resistance cassette and generate pKO-XSTS-glpXsm. *Msm* mc^2^155 was first transformed with pKO-XSTS-glpXsm and plated on 7H10 agar with hygromycin at the permissive temperature of 30 °C. Transformants were then grown at 30 °C to OD_580_=1 and plated on 7H10 agar with hygromycin, 10% sucrose, 0.2% glycerol and 0.4% glucose at the restrictive temperature of 40 °C. For complementation of Δ*glpX*_*Msm*_, we generated pGMCK-phsp60-glpX, a vector that expresses *glpX* under the control of the *hsp60* promoter and integrates into the attL5 site of the *Msm* genome. For overexpression of GPM2 in Δ*glpX*_*Msm*_, we generated pGMEK-phsp60-gpm2-FLAG (SD), an episomal vector that expresses *gpm2* (*rv3214*) with a C-terminal Flag tag under the control of the *hsp60* promoter.

Δ*glpX*Δ*gpm2* was generated by a recombineering strategy as described previously^50^. First, ∼800 bp fragments corresponding to regions upstream and downstream of *gpm2* (*rv3214*) were amplified by PCR and cloned into pDE43-XSS to flank the hygromycin resistance cassette and generate pKO-XSS-gpm2. The *gpm2* KO cassette PCR product was then produced by PCR amplification of the pKO-XSS-gpm2 zeocin cassette with the flanking *gpm2* upstream and downstream regions. *Mtb* Δ*glpX* was transformed with the recombineering vector pNit-RecET-sacB (gift from Christopher M. Sassetti) and plated on 7H10 agar with kanamycin to generate the appropriate recombineering strain: Δ*glpX*+pNit-RecET-sacB. A primary culture of Δ*glpX*+pNit-RecET-sacB grown to OD_580_=1 was diluted 25-fold in 50 ml 7H9 media containing kanamycin and glucose as the sole carbon source and grown to OD_580_=1 at 37 °C. For induction, 10 μM isovaleronitrile was added to the culture at OD_580_=1 and incubation was carried out for 8 h at 37 °C. After 8 h, 5 ml of 2 M sterile glycine was added to the culture before incubating overnight at 37 °C. After incubation, the culture was used to prepare competent cells, which were then transformed with 500 ng of the *gpm2* KO Cassette PCR product and plated on 7H10 agar containing zeocin and glucose as the sole carbon source. The genotype-validated Δ*glpX*Δ*gpm2* clone was cured of the recombineering plasmid by plating on 7H10 agar containing 10% sucrose, zeocin and glucose as the sole carbon source and then by testing for kanamycin sensitivity. Complementation of Δ*glpX*Δ*gpm2* with *glpX* was achieved using pGMCK-pTb21-glpX, which was also used to complement Δ*glpX*. Complementation of Δ*glpX*Δ*gpm2* with *gpm2* was achieved using pGMEK-phsp60-gpm2-FLAG (SD), the same vector used to complement Δ*glpX*_*Msm*_.

### FBPase activity assay

Protein lysates were prepared from 50 ml cultures in specific media at OD_580_=1. Briefly, cells were washed once in 20 mM Tris-HCl pH 7.7, and resuspended in 1 ml of the same buffer containing 0.1 μg ml^−1^ lysozyme (Sigma-Aldrich) and 1 × Roche complete, EDTA-free Protease Inhibitor Cocktail. Lysis was achieved by bead beating with 0.1 mm zirconia/silica beads three times at 4,500 r.p.m. for 30 s with samples kept on ice for 5 min between beatings. Beads and cell debris were removed by centrifugation (11,000*g*, 10 min, 4 °C) and the supernatant was passed through a 0.22 μm filter.

FBPase activity measurement was done using a previously described spectrophotometric assay that couples F6P production to conversion of NADP^+^ to NADPH, which can be detected as a change in absorbance at 340 nm (refs 23, 51). Reactions were performed in cuvettes with a 1 ml final reaction volume containing 20 mM Tris-HCl pH 7.7, 8 mM MgCl_2_, 50 mM KCl, 1 mM NADP^+^, 1 U ml^−1^ yeast G6P dehydrogenase (Sigma-Aldrich), 2.5 U ml^−1^ yeast phosphoglucoisomerase (Sigma-Aldrich) and either 50 μg ml^−1^ total protein lysate or 0.05 μg ml^−1^ purified recombinant GPM2. Reactions were incubated at 30 °C for 5 min before starting the reactions by adding FBP. Reactions were followed using a Uvikon XL UV/VIS spectrophotometer to measure absorbance at 340 nm every 30 s for at least 10 min at 30 °C. Absorbance data was collected and analysed using LabPower Jr software, version 2.06-0106S. For enzymology of GPM2, FBP concentrations were varied between 0 and 18 mM FBP, and allosteric sigmoidal kinetic parameters (*K*_m_, *k*_cat_ and *h*) were determined by a non-linear curve fitting to the three parameter Hill equation using SigmaPlot software, version 8.0.

### Immunoblot analysis

Protein lysates were prepared in the same manner as described for the FBPase activity assay. 50-100 μg total protein were separated by SDS–PAGE and then transferred to nitrocellulose membranes for probing with rabbit antisera against *Mtb* GLPX, GPM2, proteasome beta subunit (PRCB)^52^ or enolase (ENO)^10^. GLPX and GPM2 antisera were generated by Genscript using full length, 6 × His-tagged recombinant *Mtb* proteins that were produced and purified from *E. coli*. Anti-GLPX was used at 1:50 dilution. Anti-GPM2 was used at 1:1,000 dilution. Anti-PRCB was used at 1:20,000 dilution. Anti-ENO was used at 1:1,000 dilution. As secondary antibody, ECL anti-rabbit IgG, horseradish peroxidase (HRP)-linked whole antibody from donkey (GE Healthcare) was used at 1:10,000 dilution. Millipore Immobilon Western Chemiluminescent HRP Substrate or Pierce ECL Western Blotting Substrate (Thermo Scientific) were used for detection of HRP on film.

### FBPase activity purification by HPLC

Δ*glpX* protein lysate was prepared from 2 l of culture by the same method used for the FBPase activity assay. Four steps of liquid chromatography were done to purify the FBPase activity using an ÄKTAFPLC liquid chromatography system (GE Healthcare). After each step, active fractions were identified using the described FBPase activity assay and then pooled for further purification. All HPLC columns were obtained from GE Healthcare. At a minimum, the Buffer A used in each purification step consisted of 20 mM Tris-HCl pH 7.7, 8 mM MgCl_2_, 10% glycerol and 10 mM β-mercaptoethanol. First, anion exchange chromatography was performed with a 5 ml HiTrap Q Sepharose FF column using a gradient from 0 to 6 M NaCl over 25 column volumes. Next, the pooled active fractions were diluted 1:1, with 2 M ammonium sulfate and hydrophobic interaction chromatography was performed with a 1 ml HiTrap Phenyl Sepharose FF (high sub) column using a gradient from 1 to 0 M ammonium sulfate over 20 column volumes. Active fractions were pooled and concentrated using a Microcon YM-3 (3000 MWCO) centrifugal filter device (Millipore) before performing size-exclusion chromatography with a Superose 6 10/300 GL column using Buffer A with 650 mM NaCl as the mobile phase. Biorad Gel Filtration Standard (#151-1901) was used to calibrate the Superose 6 10/300 GL column. Last, a second, higher resolution anion exchange chromatography was done with a Mono Q 5/50 GL column using a gradient from 0.1 to 0.4 M NaCl over 25 column volumes. The final active fractions were run on a 15% SDS–PAGE gel and stained using the Invitrogen Silver Quest staining kit. A protein band whose intensity matched the FBPase activity profile of the active fractions was cut, processed and analysed by liquid chromatography-tandem mass spectroscopy (LC-MS/MS) by the Proteomics Resource Center at The Rockefeller University. SDS–PAGE-separated and silver-stained proteins were in-gel trypsinized as described elsewhere^53^. Extracted peptides were measured by reversed phase nano-LC-MS/MS (NCS3500RS Nano and Q-Exactive, Thermo Scientific). Tandem MS data was extracted using ProteomeDiscoverer v1.3 (Thermo, Bremen, Germany) and queried against UniProts *Mtb* H37Rv database using MASCOT 2.3 (Matrixscience, London, UK). Peptides with a Percolator-based false discovery^54^ rate of 1% or better were reported.

### Expression and purification of recombinant GPM2

pET300-NT-gpm2, a vector for inducible expression of an N-terminal 6 × His-tagged GPM2 in *E. coli*, was constructed using Gateway Cloning Technology. *Gpm2* (*rv3214*) was cloned using primers that allow for recombination into the Champion pET300/NT-DEST vector: 5′-GGGGACAAGTTTGTACAAAAAAGCAGGCTTGGGCGTGCGCAACCACCGATTGCTAC-3′ and 5′-GGGGACCACTTTGTACAAGAAAGCTGGGTTGTGCGCTCACCCGGCTGCGATCGGC-3′. pET300-NT-gpm2 was transformed into *E. coli* BL21 (DE3) (from Invitrogen) and plated on Luria-Bertani (LB) agar containing 100 μg ml^−1^ carbenicillin. Transformants were scraped from the plates into 5 ml LB broth and used to inoculate 2 l LB broth with 100 μg ml^−1^ carbenicillin. Cultures were incubated at 37 °C with shaking until they reached OD_600_=0.6. At this point, protein expression was induced by adding a final concentration of 0.1 mM isopropyl-D-1-thiogalactopyranoside to the cultures and incubation was continued overnight at 18 °C. Cultures were then centrifuged at 4,000*g* for 20 min at 4 °C. Cell pellets were washed once with 30 ml PBS and resuspended in 20 ml Buffer A (40 mM Tris-HCl, 400 mM NaCl, 10% glycerol, 1 mM β-mercaptoethanol, 1 × Roche complete and EDTA-free Protease Inhibitor Cocktail pH 8.0). The resuspension was then subjected to sonication on ice using a Virtis Virsonic 600 sonicator (Output Setting 6, six 30 s pulses with 50 s breaks) and centrifuged at 16,000*g* for 1 h at 4 °C to remove cell debris. The supernatant was clarified using a 0.45 μm polyvinylidene difluoride filter and loaded onto a His Trap HP 5 ml column (GE Healthcare) that had been pre-equilibrated with Buffer A. The column was washed with 20 column volumes of Buffer A with 50 mM imidazole. Bound 6 × His-tagged GPM2 was eluted from the column with 3 column volumes of Buffer A with 500 mM imidazole. Purity of the sample was assessed by SDS–PAGE. The sample was dialysed against Buffer A without glycerol using a Thermo Slide-a-lyzer Cassette (12 ml capacity, 10 kDa cut off) and then concentrated using an Amicon Ultra centrifugal device (Regenerated cellulose, 3000 MWCO). Part of the final sample was stored with 50% glycerol at −20 °C for FBPase activity assays or flash frozen with liquid nitrogen and stored at −80 °C for use in antibody generation.

### Metabolomics

Metabolite samples were separated and detected on an Agilent Accurate Mass 6220 TOF coupled to an Agilent 1200 Liquid Chromatography system using a Cogent Diamond Hydride Type C column (Microsolve Technologies) using solvents and configuration as described before^47^. To quantify metabolites, standard curves were generated using authentic chemical compounds that were spiked into mycobacterial lysates. The following chemical compounds were used to quantify metabolites: D-G6P (for hexose-phosphate), D-S7P, D-FBP (for hexose-biphosphate), D-glyceraldehyde 3-phosphate (for triose-phosphate), phosphoenolpyruvic acid, pyruvic acid and L-aspartic acid. Metabolite concentrations were normalized to biomass based on measurement of residual peptide content in individual samples using the Pierce BCA Protein Assay kit.

### Mouse infection model of *Mtb*

Aerosol infection of 7-week-old female C57BL/6 mice (Jackson Laboratory) was performed using an inhalation exposure system from Glas-Col, and early log phase *Mtb* cultures were prepared as single-cell suspensions in PBS to deliver 100–200 bacilli per mouse. Four mice were killed per strain per time point. Serial dilutions of lung and spleen homogenates were plated on appropriate 7H10 agar plates as described in the ‘strains and culture conditions’ section. The left lobe of the mouse lungs was fixed in 10% formalin in PBS and used for histopathology staining with haematoxylin and eosin. Procedures involving mice were reviewed and approved by the Institutional Animal Care and Use Committee of Weill Cornell Medical College.

## Additional information

**How to cite this article:** Ganapathy, U. *et al.* Two enzymes with redundant fructose bisphosphatase activity sustain gluconeogenesis and virulence in *Mycobacterium tuberculosis*. *Nat. Commun.* 6:7912 doi: 10.1038/ncomms8912 (2015).

## Supplementary Material

Supplementary Information Supplementary Figures 1-9 and Supplementary Tables 1-3

## Figures and Tables

**Figure 1:**
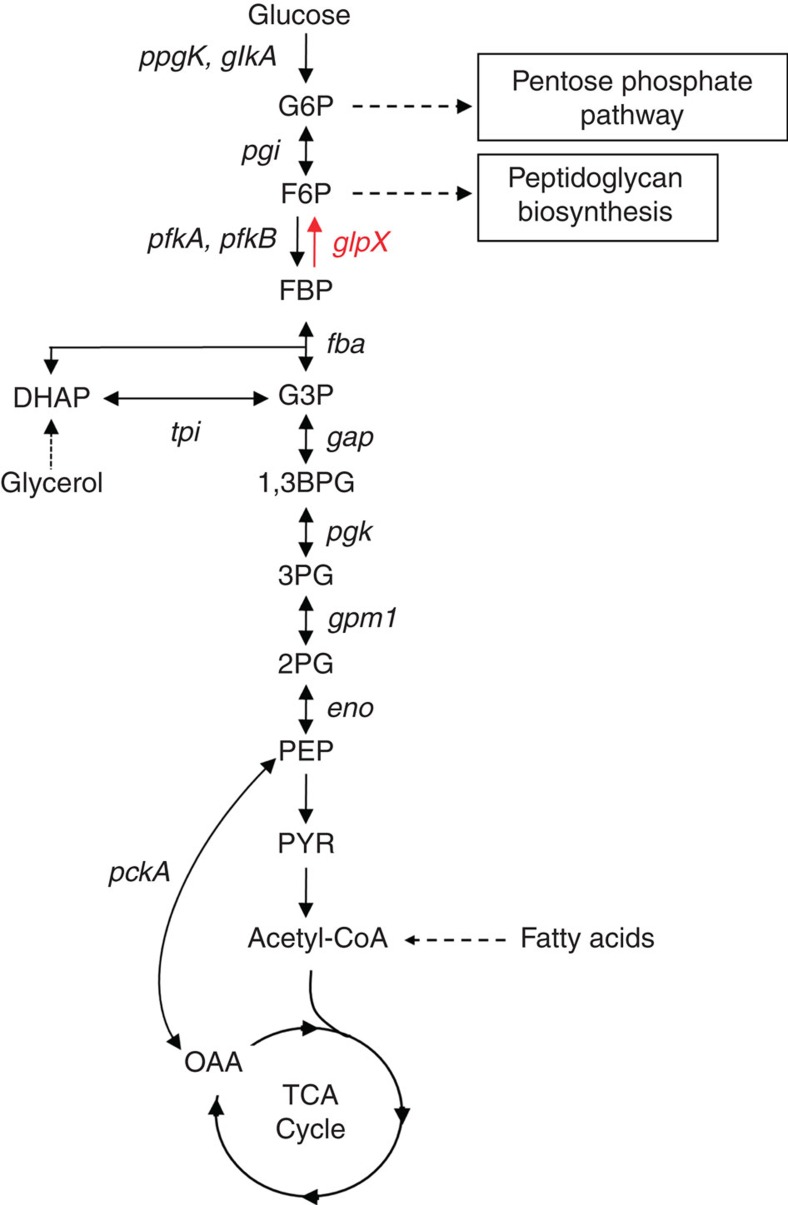
Metabolic schematic of glycolysis and gluconeogenesis. *GlpX* (*rv1099c*) encodes the only known FBPase in the *Mtb* genome and controls the rate-limiting step of gluconeogenesis. Other enzymes of the gluconeogenesis pathway are encoded by *pckA* (phosphoenolpyruvate carboxykinase), *eno* (enolase), *gpm1* (phosphoglycerate mutase), *pgk* (phosphoglycerate kinase), *gap* (glyceraldehyde 3-phosphate dehydrogenase), *tpi* (triose phosphate isomerase), *fba* (fructose bisphosphate aldolase) and *pgi* (glucose 6-phosphate isomerase). Also shown are *ppgK* (polyphosphate glucokinase), *glkA* (glucokinase) and *pfkA* and *pfkB* (phosphofructokinases), which encode glycolysis-specific enzymes. 1,3BPG, 1,3-bisphosphoglycerate; 2PG, 2-phosphoglycerate; 3PG, 3-phosphoglycerate; DHAP, dihydroxyacetone phosphate; F6P, fructose 6-phosphate; FBP, fructose 1,6-bisphosphate; G3P, glyceraldehyde 3-phosphate; G6P, glucose 6-phosphate; OAA, oxaloacetate; PEP, phosphoenolpyruvate; PYR, pyruvate.

**Table 1:** Comparison of GPM2 and GLPX FBPase activity properties.

	**GPM2**	**GLPX**
Enzymatic kinetics model	Allosteric sigmoidal	Michaelis–Menten
*K*_m_	5.51 mM	44 μM
*k*_cat_	1.87 × 10^2^ s^−1^	1.0 s^−1^
h	3.02	—
*k*_cat_/*K*_m_	3.39 × 10^4^ s^−1^ M^−1^	2.27 × 10^4^ s^−1^ M^−1^

FBPase, fructose bisphosphatase.

GPM2 enzymology determined experimentally using recombinant His-tagged GPM2. GLPX enzymology from published literature^23^.
