# Supplementary material for: Two enzymes with redundant fructose bisphosphatase activity sustain gluconeogenesis and virulence in Mycobacterium tuberculosis
Source: Nat Commun. 2015 Aug 10;6:7912. doi: 10.1038/ncomms8912 (PMC4535450; doi:10.1038/ncomms8912)
Supplement: Supplementary Information — Supplementary Figures 1-9 and Supplementary Tables 1-3 [file ncomms8912-s1.pdf]

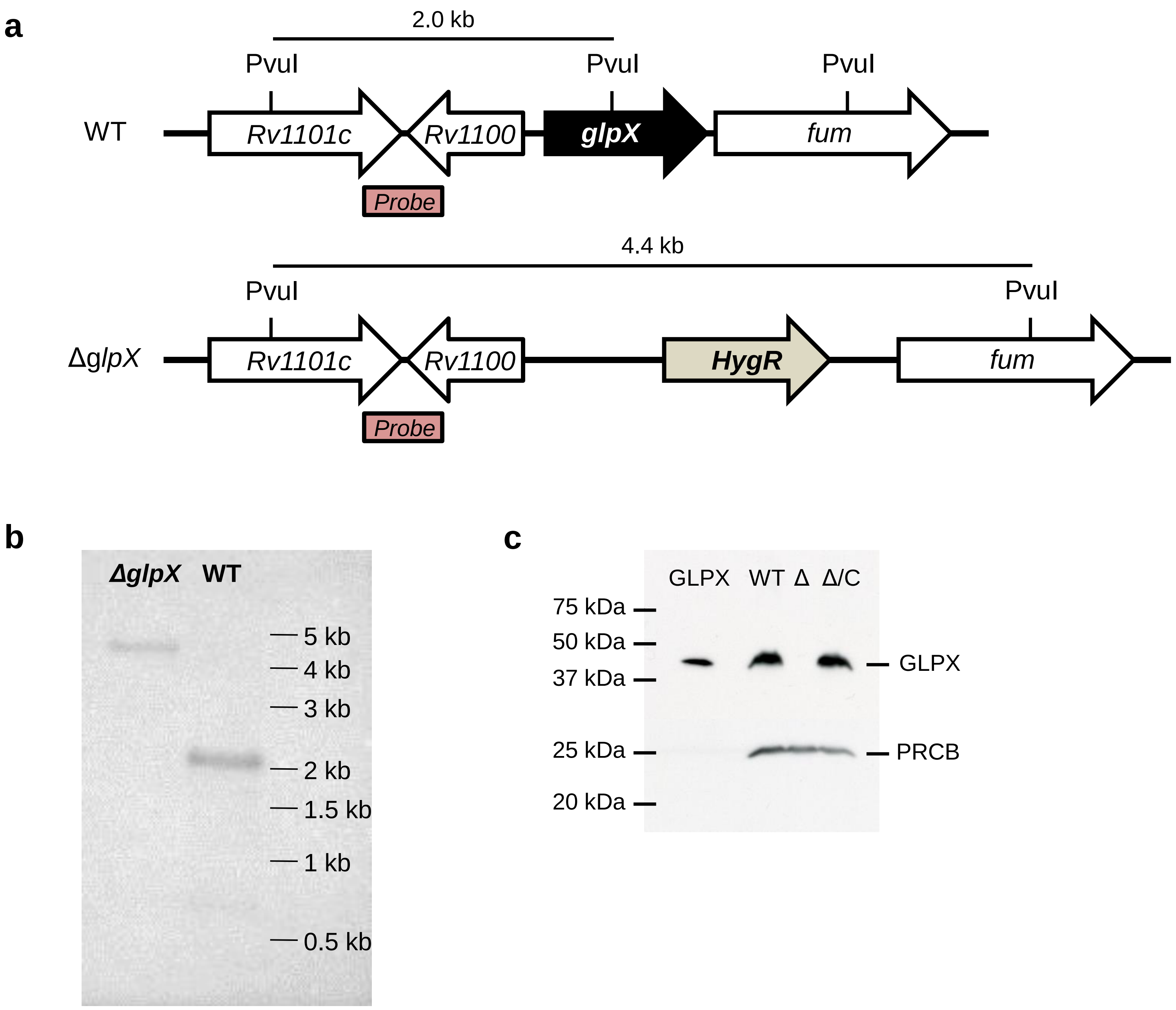

Supplementary Fig. 1. Generation and Validation of Δ*glpX*. (**a**) Strategy for deleting *glpX* (*Rv1099c*) by homologous recombination and validating Δ*glpX* candidates by PvuI digest and southern blot analysis. (**b**) Southern blot analysis of WT *Mtb* and Δ*glpX* where genomic DNA was digested with PvuI. (**c**) Western blot analysis of WT *Mtb* (WT), Δ*glpX* (Δ), and complemented strain Δ*glpX*/C (Δ/C) cell lysates with anti-GLPX antibody. Molecular weight of GLPX is 38.09 kDa. Recombinant His-tagged GLPX (GLPX, 39.96 kDa) was run as a positive control. Anti-PRCB was used to confirm equal loading.

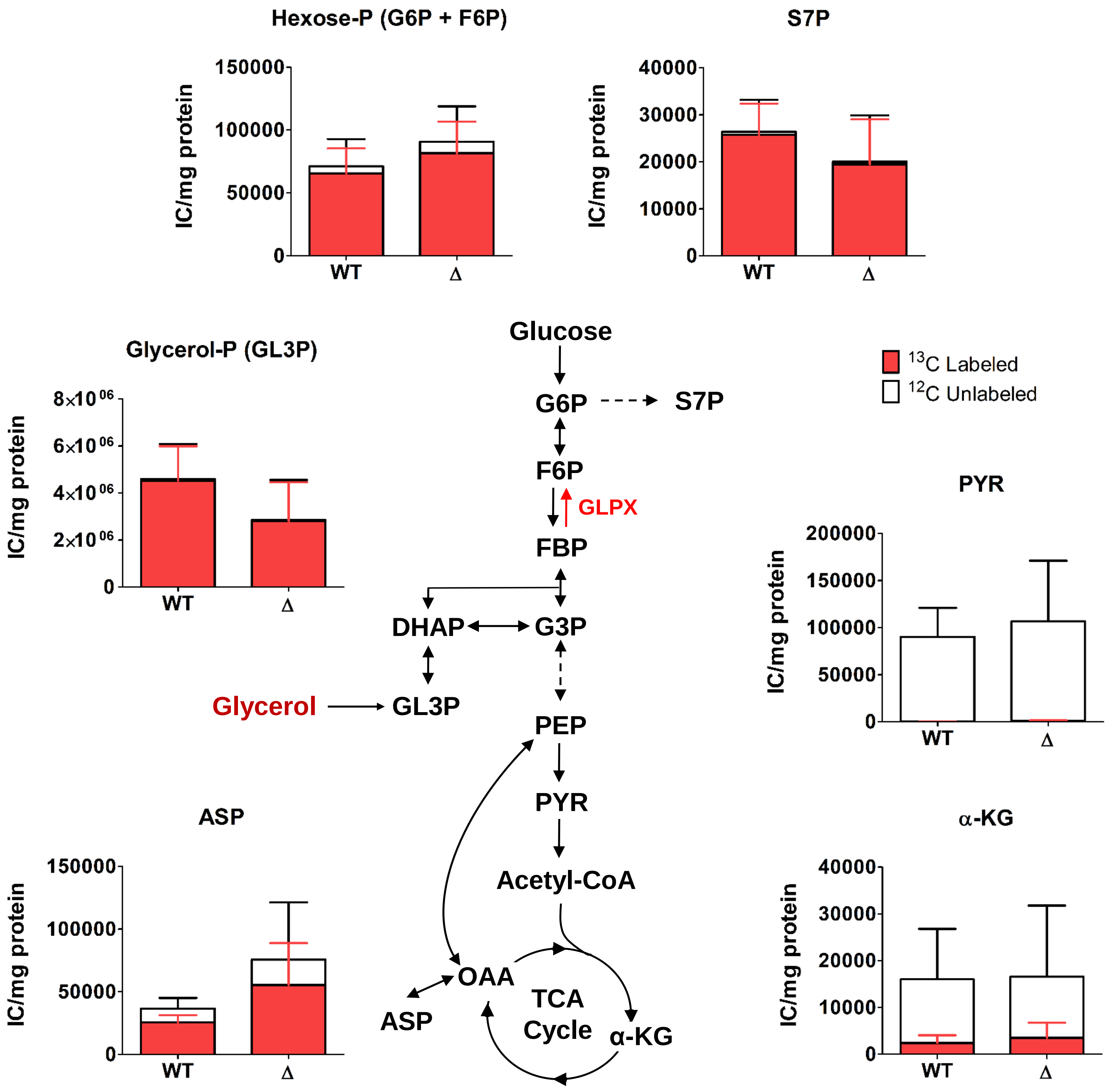

Supplementary Fig. 2. Gluconeogenesis is not strictly dependent on GLPX. Abundance and ^{13}C labeling of metabolites in WT *Mtb* and Δ*glpX* (Δ). Bacteria were grown on glycerol-containing plates for 5 days and then transferred to U-^{13}C glycerol-containing plates for an additional 16h prior to harvesting. Data are means ± standard deviation of three biological replicates and are representative of two independent experiments. Data are reported as ion counts (IC) per mg total protein. *0.01<*P*≤0.05, **0.001<*P*≤0.01, ****P*≤0.001 by Student's t-test. G6P: glucose 6-phosphate, S7P: sedoheptulose 7-phosphate, F6P: fructose 6-phosphate, FBP: fructose 1,6-bisphosphate, DHAP: dihydroxyacetone phosphate, G3P: glyceraldehyde 3-phosphate, GL3P: glycerol 3-phosphate, PEP: phosphoenolpyruvate, PYR: pyruvate, OAA: oxaloacetate, ASP: aspartate α-KG: α-ketoglutarate.

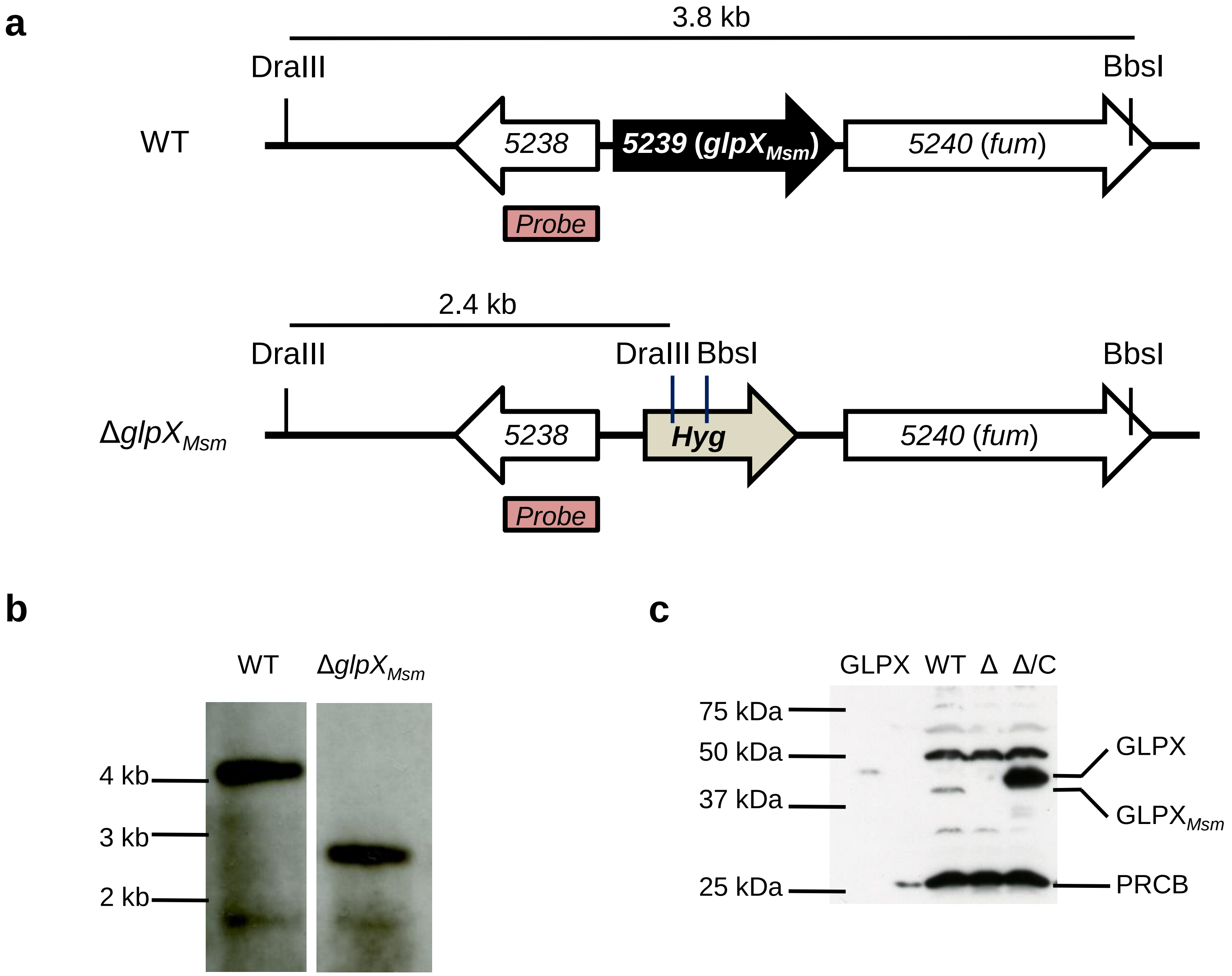

Supplementary Fig. 3. Generation and Validation of Δ*glpX*$_{Msm}$. (**a**) Strategy for deleting *msmeg_5239* (*glpX* homolog in *M. smegmatis*) by homologous recombination and validating Δ*glpX*$_{Msm}$ candidates by DraIII and BbsI double digest and southern blot analysis. (**b**) Southern blot analysis of WT *Msm* and Δ*glpX*$_{Msm}$ where genomic DNA was digested with DraIII and BbsI. (**c**) Western blot analysis of WT *Msm* (WT), Δ*glpX*$_{Msm}$ (Δ), and complemented strain Δ*glpX*$_{Msm}$/C (*glpX*) (Δ/C) cell lysates with anti-GLPX antibody. Molecular weight of GLPX$_{Msm}$ is 35.87 kDa. Molecular weight of GLPX is 38.09 kDa. Recombinant His-tagged GLPX (GLPX, 39.96 kDa) was run as a positive control. Anti-PRCB was used to confirm equal loading.

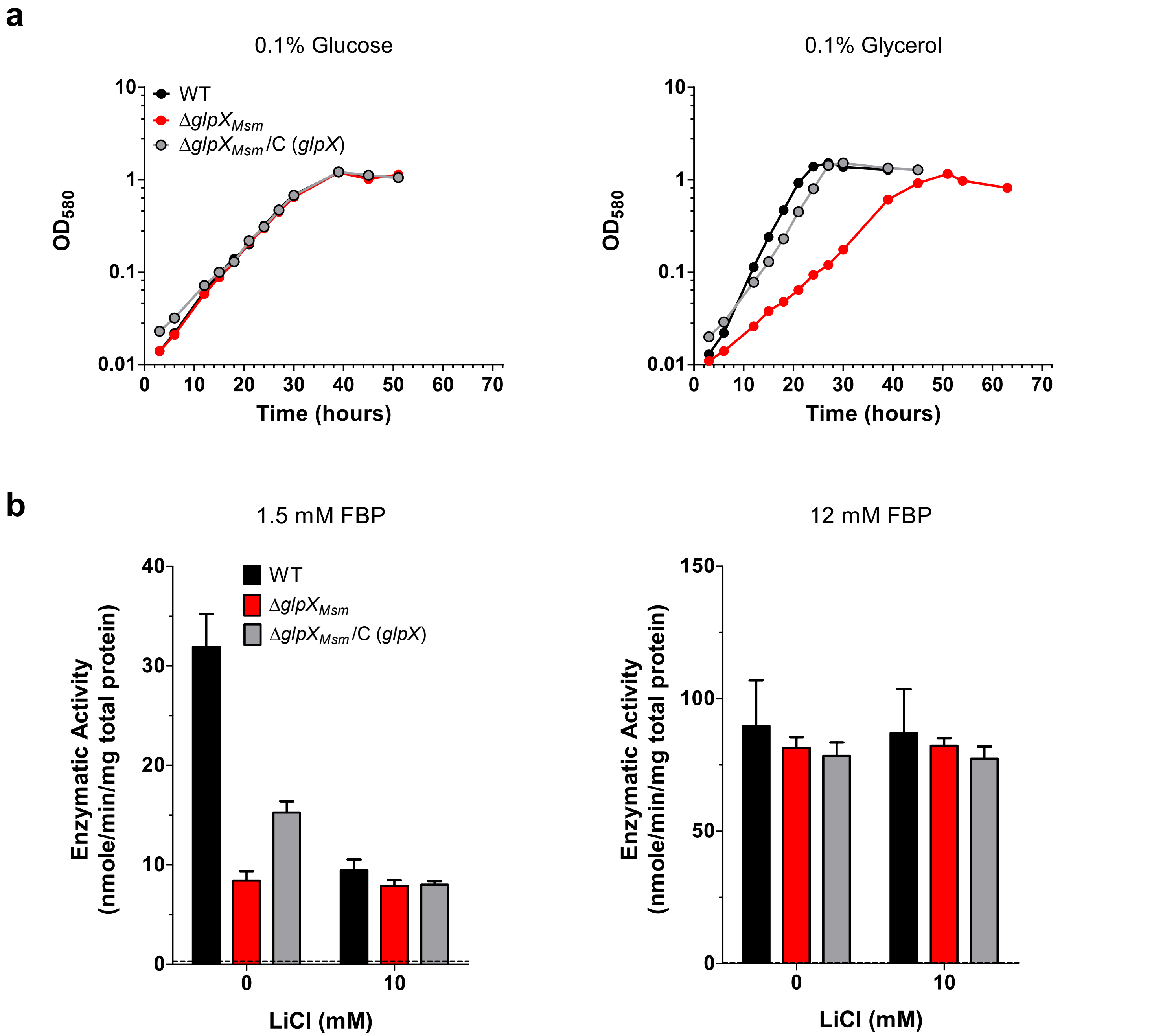

Supplementary Fig. 4. Δ*glpX*$_{Msm}$ can grow on gluconeogenic carbon sources and has detectable FBPase activity. (**a**) Growth of WT *Msm* (black), Δ*glpX*$_{Msm}$ (red) and complemented strain Δ*glpX*$_{Msm}$/C (*glpX*) (gray) in 7H9 media containing 0.1% glucose or 0.1% glycerol as the sole carbon source. Data are representative of three independent experiments. (**b**) FBPase activity of WT *Msm* (black), Δ*glpX*$_{Msm}$ (red) and complemented strain Δ*glpX*$_{Msm}$/C (*glpX*) (gray) cell lysates in the absence or presence of lithium chloride using 1.5 mM F-1,6BP or 12 mM F-1,6BP as substrate. Dashed line indicates limit of detection. Data are means ± standard deviation of three biological replicates.

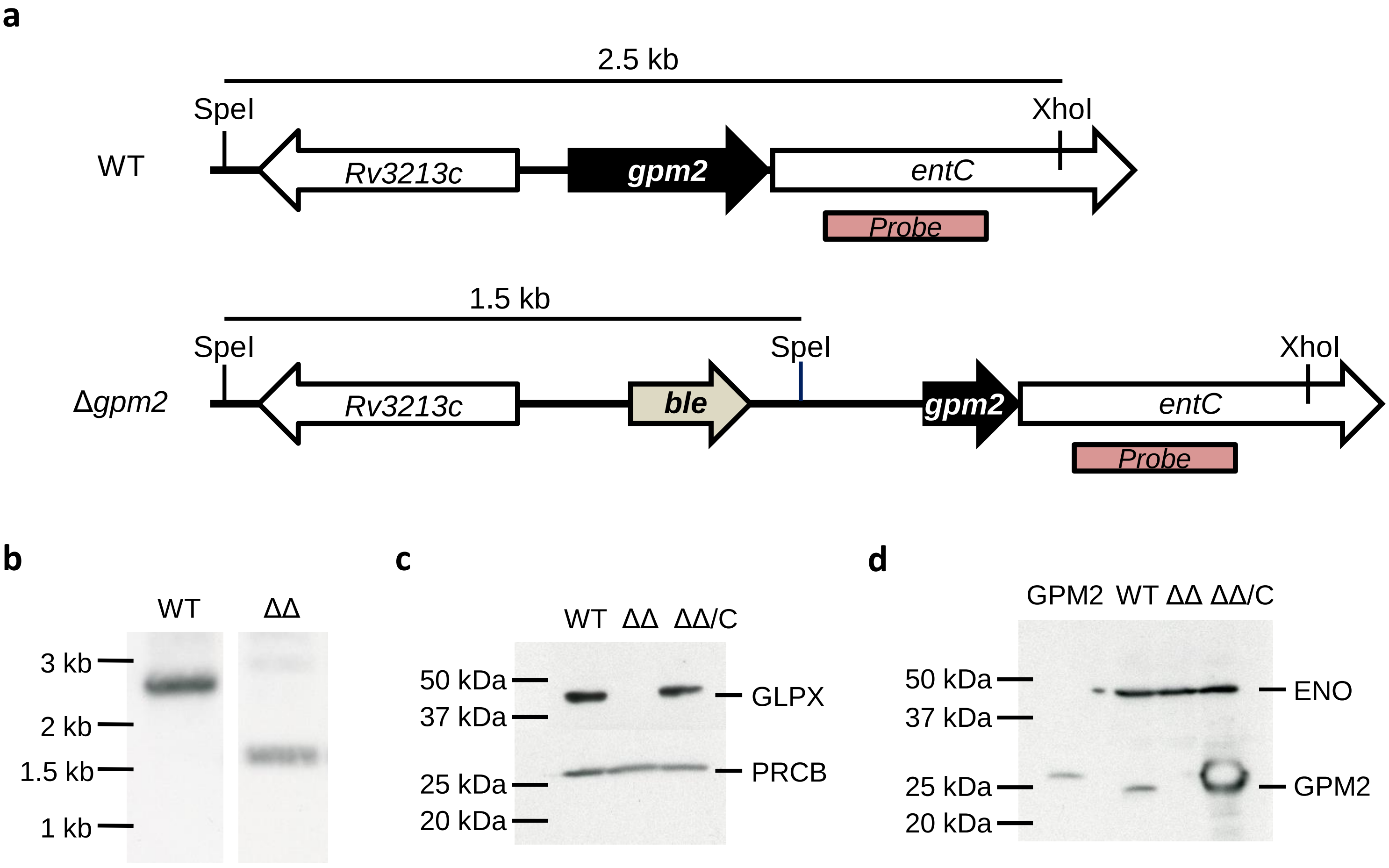

Supplementary Fig. 5. Generation and Validation of *Mtb* Δ*glpX*Δ*gpm2*. (**a**) Strategy for deleting *gpm2* (*Rv3214*) by homologous recombination and validating Δ*glpX*Δ*gpm2* candidates by SpeI and XhoI double digest and southern blot analysis. (**b**) Southern blot analysis of WT *Mtb* and Δ*glpX*Δ*gpm2* where genomic DNA was digested with SpeI and XhoI. (**c**) Western blot analysis of WT *Mtb* (WT), Δ*glpX*Δ*gpm2* (ΔΔ), and complemented strain Δ*glpX*Δ*gpm2*/C (*glpX*) (ΔΔ/C) cell lysates with anti-GLPX antibody. Molecular weight of GLPX is 38.09 kDa. Anti-PRCB was used to confirm equal loading. (**d**) Western blot analysis of WT *Mtb* (WT), Δ*glpX*Δ*gpm2* (ΔΔ), and complemented strain Δ*glpX*Δ*gpm2*/C (*gpm2*) (ΔΔ/C) cell lysates with anti-GPM2 antibody. Molecular weight of GPM2 is 21.95 kDa. Recombinant His-tagged GPM2 (GPM2, 23.85 kDa) was run as a positive control. Anti-ENO was used to confirm equal loading.

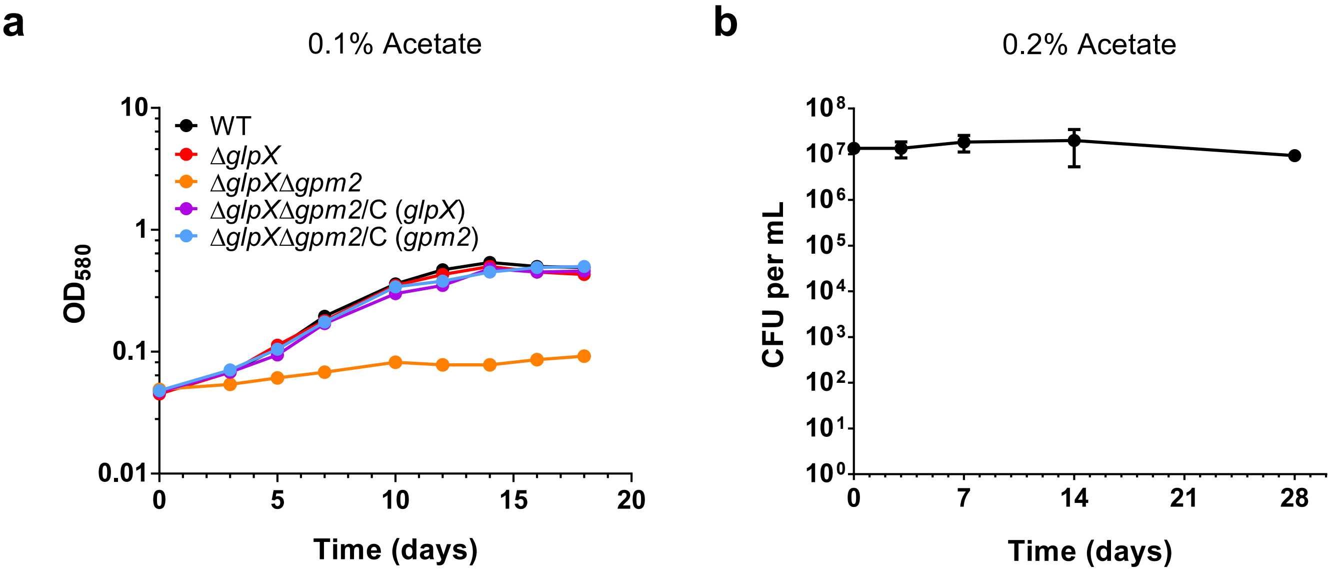

Supplementary Fig. 6. *Mtb ΔglpXΔgpm2* does not grow on fatty acids as a sole carbon source but remains viable. (**a**) Growth of WT Mtb (black), Δ*glpX* (red), Δ*glpX*Δ*gpm2* (orange) and complemented strains Δ*glpX*Δ*gpm2*/C (*glpX*) (purple) and Δ*glpX*Δ*gpm2*/C (*gpm2*) (blue) in Sauton's minimal media containing 0.1% acetate. Data are representative of three independent experiments. (**b**) Survival of Δ*glpX*Δ*gpm2* in Sauton's minimal media containing 0.2% acetate. Data represent the mean CFU/mL of three biological replicates at different time points.

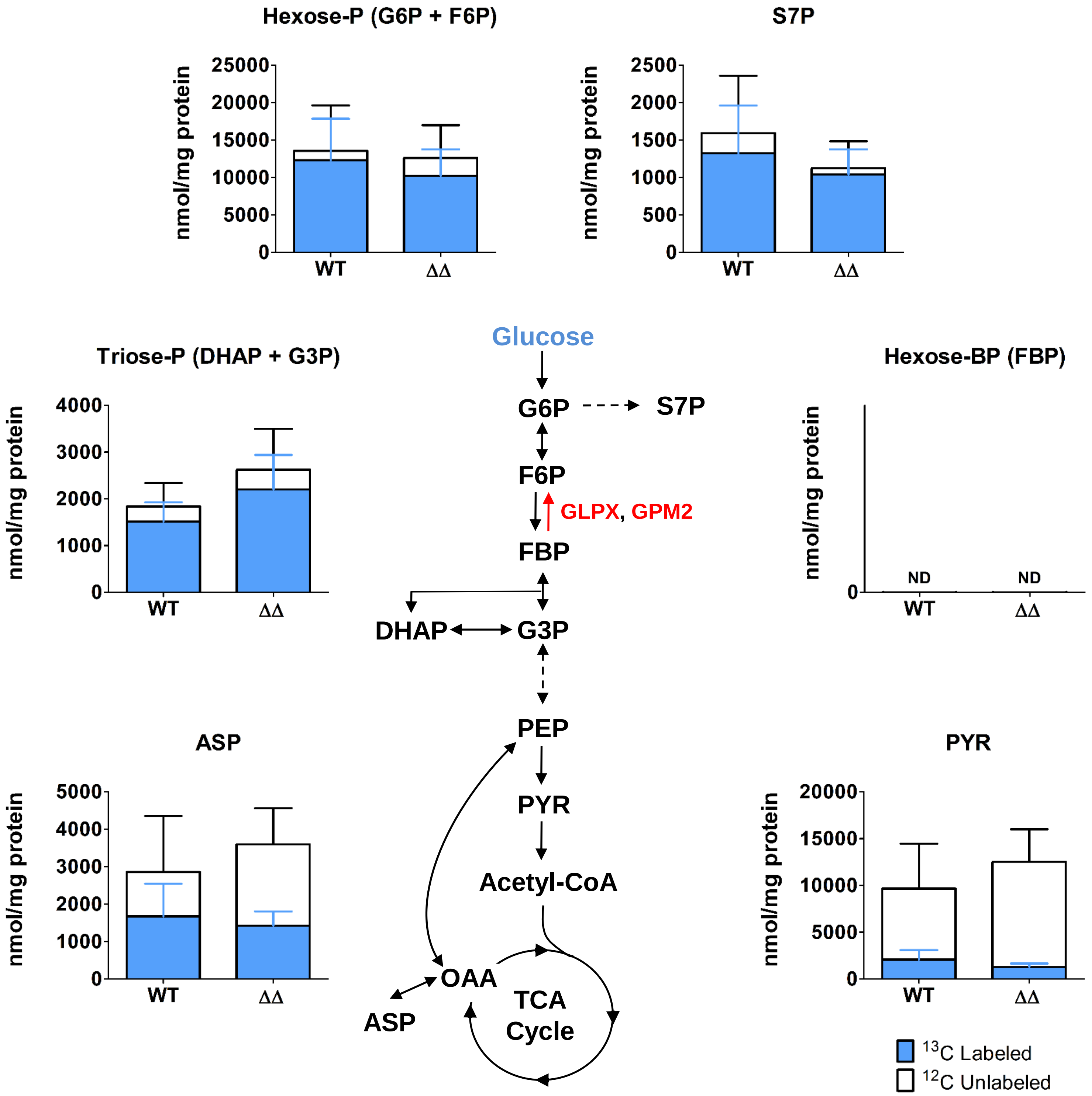

Supplementary Fig. 7. Glycolysis is unperturbed in *Mtb* Δ*glpX*Δ*gpm2*. Abundance and ^{13}C labeling of metabolites in WT *Mtb* and Δ*glpX*Δ*gpm2* (ΔΔ). Bacteria were grown on glucose-containing plates for 5 days and then transferred to U-^{13}C glucose-containing plates for an additional 24h prior to harvesting. Data are means ± standard deviation of three biological replicates and are representative of two independent experiments. *0.01<*P*≤0.05, **0.001<*P*≤0.01, ****P*≤0.001 by Student's t-test. ND indicates that the recovered level of FBP was below the limit of detection (3.13 μM). G6P: glucose 6-phosphate, S7P: sedoheptulose 7-phosphate, F6P: fructose 6-phosphate, FBP: fructose 1,6-bisphosphate, DHAP: dihydroxyacetone phosphate, G3P: glyceraldehyde 3-phosphate, PEP: phosphoenolpyruvate, PYR: pyruvate, OAA: oxaloacetate, ASP: aspartate.

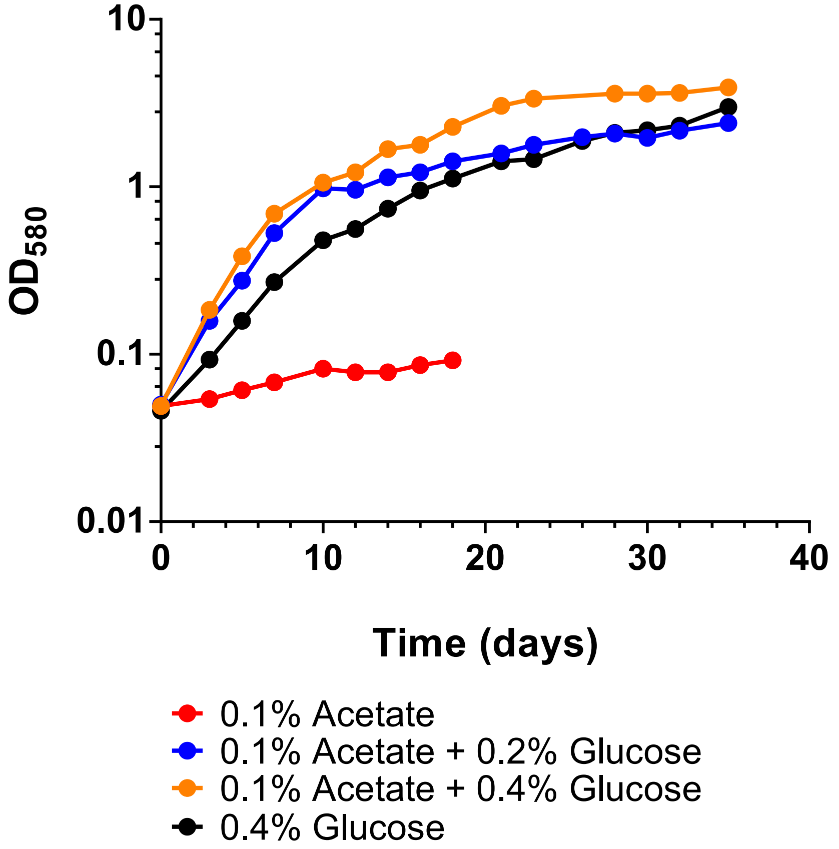

Supplementary Fig. 8. Growth of *Mtb* Δ*glpX*Δ*gpm2* on glucose is enhanced by the presence of acetate. Growth of Δ*glpX*Δ*gpm2* in Sauton's minimal media containing 0.1% acetate (red), 0.1% acetate and 0.2% glucose (blue) 0.1% acetate and 0.4% glucose (orange) or 0.4% glucose (black) Data are representative of three independent experiments.

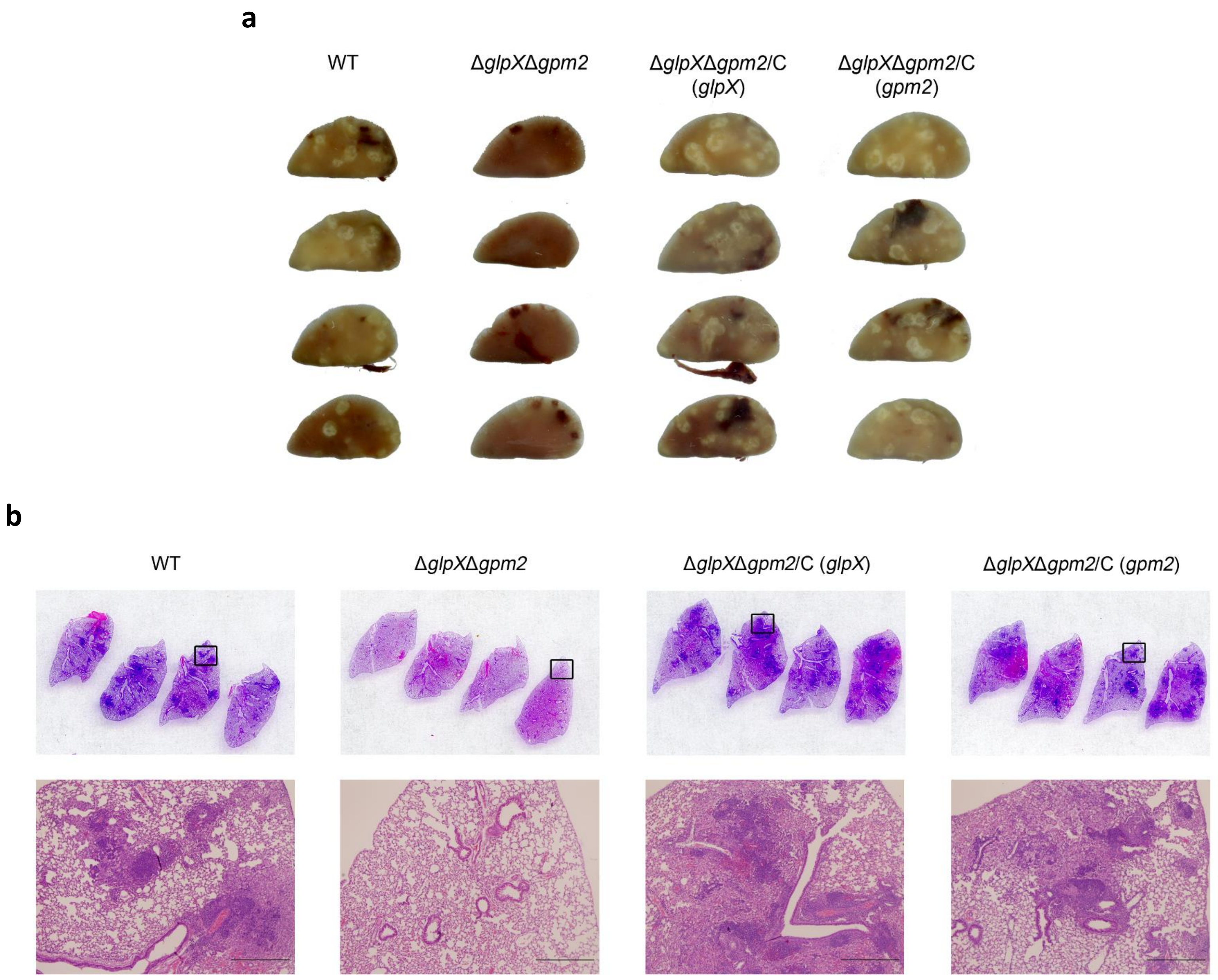

Supplementary Fig. 9. *Mtb* Δ*glpX*Δg*pm2* is attenuated *in vivo* – Lung histopathology. (**a**) Gross pathology of left lung lobes from infected C57BL/6 mice at Day 56 post-infection. (**b**) Hemotoxylin and eosin staining of sections from left lung lobes of infected C57BL/6 mice at Day 56 post-infection. Top row of images show sections from four mice per group. Black box indicates region magnified in the bottom row of images. The scale bar is 10 μm.

Purification Step	Protein Concentration (mg/mL)	Total Protein (mg)	Activity (nmol/min)	Specific Activity (nmol/min/mg protein x10^3)	Fold Enrichment
Total Protein Lysate	1.49	14.91	5320	0.357	1.00
Anion Exchange (Q Sepharose)	0.338	1.39	1920	1.38	3.87
Hydrophobic Interaction (Phenyl Sepharose)	0.0546	0.339	1030	3.04	8.50
Gel Filtration (Superose 6)	0.0136	0.00953	181	19.0	53.27
Anion Exchange (Mono Q)	≤0.0025*	≤0.00125*	29.1	≥23.3*	≥65.13*

Supplementary Table 1. Purification of second FBPase activity from Δ*glpX* cell lysate. Four purification steps were done in total. Initial FBPase activity and protein concentration for the total lysate sample are shown. After the anion exchange (Q Sepharose), hydrophobic interaction and gel filtration steps, the active fractions were pooled and the FBPase activity and protein concentration of were measured. After the anion exchange (Mono Q) step, the FBPase activity and protein concentration were measured for just the peak activity fraction (Fraction B15). Total protein, specific activity and fold enrichment of activity were determined for each sample accordingly. Asterisks indicate that the protein concentration of the anion exchange (Mono Q) sample was below the limit of detection of the protein measurement assay used (0.0025 mg/mL). As a result, total protein, specific activity and fold enrichment values for this step are presented as minimum values based on the limit of detection of the protein measurement assay.

Peptide Sequence	MH^+ (Da)
HTGGTEVELTDTGR	1472.70
HGETAWSTLGR	1214.59
WVQLPLAEGSR	1255.68
ADSAVALALEHMSSR	1573.76
TQAELAGQLLGELELDDPIVICSPR	2737.40
QLAVLGLTGHPQPIAAG	1642.92
DVLFVSHGHFSR	1400.71
LAGLTVNEVTGLLAEWDYGSYEGLTTPQIR	3266.67

Supplementary Table 2. GPM2 peptides identified by LC-MS/MS of 25 kDa gel band from Figure 2.

Name	Sequence	Use in This Study
JM17	5’ TATACTTAAGCGGCGTTGCTCTGGGTCAAGCTCAG 3’	Δ*glpX* Knockout Vector
JM18	5’ AATACCTAGGGGCCAGGTTGCGGTCCGGGGCTTCC 3’	Δ*glpX* Knockout Vector
JM19	5’ TATAAAGCTTCACACCCACGACACACAAGGAACCC 3’	Δ*glpX* Knockout Vector
JM20	5’ ATATATGCATGGCGGCCAATGCGTCGTGCAGCTGC 3’	Δ*glpX* Knockout Vector
UG1	5’ ACCACGCACCAATGCTCTAC 3’	Δ*glpX* Southern Blot Probe
UG2	5’ CGGTGGTCAAGACCTGGTTC 3’	Δ*glpX* Southern Blot Probe
KO-glpXsm-attB1r	5’ GGGGACTGCTTTTTTGTACAAACTTGTGCCTCGGTTACCCGGACAAGTTC 3’	Δ$glpX_{Msm}$ Knockout Vector
KO-glpXsm-attB4	5’ GGGGACAACTTTGTATAGAAAAGTTGGTACTCGCTGGTGTCCGAGTTACG 3’	Δ$glpX_{Msm}$ Knockout Vector
KO-glpXsm-attB2	5’ GGGGACAGCTTTCTTGTACAAAGTGGACACCAACAAACAAGGGACGCAACAG 3’	Δ$glpX_{Msm}$ Knockout Vector
KO-glpXsm-attB3	5’ GGGGACAACTTTGTATAATAAAGTTGTTGACGAGCACCTCGACGACCTTG 3’	Δ$glpX_{Msm}$ Knockout Vector
UG3	5’ TACGAGCTGGGCTTCATCGAC 3’	Δ$glpX_{Msm}$ Southern Blot Probe
UG4	5’TGGGATACTGGCAGGCATGAC 3’	Δ$glpX_{Msm}$ Southern Blot Probe
clo-glpX-attB2	5’ GGGGACAGCTTTCTTGTACAAAGTGGAAGGAGGACCAACCATGACAGCTGAGG GATCCGGTT 3’	*glpX* Complementation Vector
JM33	5’ GGGGACAACTTTGTATAATAAAGTTGCTTAGGGCAATGGGTACACGGCGCTGCT GTCGC 3’	*glpX* Complementation Vector
UG17-SD	5’ GGGGACAGCTTTCTTGTACAAAGTGGAAGGAGGTGTCGGTATGGGCGTGCGCA ACCACCG 3’	*gpm2* Complementation Vector
UG18	5’ CTTATCGTCATCGTCCTTGTAGTCCCCGGCTGCGATCGGCTGCGGATGACC 3’	*gpm2* Complementation Vector
OE-gpm2-attB1 (UG)	5’ GGGGACAAGTTTGTACAAAAAAGCAGGCTTGGGCGTGCGCAACCACCGATTGC TAC 3’	6xHis-Tagged GPM2
OE-gpm2-attB2r	5’ GGGGACCACTTTGTACAAGAAAGCTGGGTTGTGCGCTCACCCGGCTGCGATCG GC 3’	6xHis-Tagged GPM2
KO-gpm2-attB4	5’ GGGGACAACTTTGTATAGAAAAGTTGGCGGTCGGCGACATCGAGCAACACG 3’	Δ*gpm2* Knockout Vector
KO-gpm2-attB1r	5’ GGGGACTGCTTTTTTGTACAAACTTGCCGACAGATTGTGCCCGACGACATC 3’	Δ*gpm2* Knockout Vector
KO-gpm2-attB2	5’ GGGGACAGCTTTCTTGTACAAAGTGGTGGACGCACGGCTGCCCAGCTGGAG 3’	Δ*gpm2* Knockout Vector
KO-gpm2-attB3	5’ GGGGACAACTTTGTATAATAAAGTTGTGAGGTCGACGAGATAGCCGTAAGC 3’	Δ*gpm2* Knockout Vector
UG20	5’ ATACTGTTGGGCGCGTTGCCTTTC 3’	Δ*gpm2* Southern Blot Probe
UG21	5’ TTGGCCGAACTGGCTAGTGC 3’	Δ*gpm2* Southern Blot Probe

Supplementary Table 3. List of primers used in this study.
